# One welfare: bibliometric review of world literature

**DOI:** 10.3389/fvets.2025.1627981

**Published:** 2025-08-25

**Authors:** Sara Platto, Simona Normando, Agathe Serres, Xavier Manteca, Deborah Temple

**Affiliations:** ^1^Department of Biotechnology, College of Life Sciences, Jianghan University, Wuhan, China; ^2^Department of Comparative Biomedicine and Food Sciences, Padova University, Padova, Italy; ^3^Sanya Key Laboratory of Marine Mammal and Marine Bioacustics, Institute of Deep-Sea Sciences and Engineering, Chinese Academy of Sciences, Sanya, China; ^4^Department of Animal and Food Sciences, College of Veterinary Sciences, Universitat Autonoma de Barcelona, Barcelona, Spain; ^5^AWEC, Edifici Eureka, Campus de la Universitat Autonoma de Barcelona, Barcelona, Spain

**Keywords:** one welfare, animal welfare, human welfare, environmental welfare, human-animal interaction, animal management

## Abstract

The One Welfare framework emphasizes the interconnection between animal, human, and environmental well-being, extending One Health principles to address broader welfare dimensions. Despite its relevance, One Welfare remains underexplored. This study investigates global research trends and thematic priorities in One Welfare literature published from 2013 to 2024. A bibliometric review was conducted using PubMed, Elsevier, Springer, Web of Science, Scopus, and CABI databases. A literature search was conducted using keywords translated into five of the world’s most widely spoken languages: Hindi, Chinese, Spanish, English, and French. A total of 111 publications were identified and categorized into four main domains—Policy, Governance, Economy (PGE); Applied Human-Animal Sciences (AHAS); Societal, Economic, Environmental Dimensions (SEED); and Human-Animal Bond and Mental Health (HAB-MH)—and eight subcategories: Legal Framework and Economy (LFE); Education and Philosophy (EP); Sustainable Resource Management (SRM); Traditional Knowledge and Societal Impact (TKSI); Animal Management (AM); Human-Animal Diseases (HAD); Human-Animal Interaction (HAI); and Psychology (PSY). The analysis also considered animal types—companion animals (CA), production animals (PA), wild animals (WA), working/sport animals (WS), and general (GE)—and divided data into two time periods (2013–2018, 2019–2024). Most publications (78) emerged after 2018, with Animal Management (AM) subcategory as the dominant theme, particularly in relation to PA reflecting their significance in food security. Human-Animal Interaction (HAI) was the second most represented theme among the subcategories, particularly in relation to CA underlining their significance in human lives. Conversely, wild animals (WA), climate change, and working/sport animals (WS) remain underrepresented. Education and Philosophy was the least addressed subcategory, exposing a critical gap in integrating One Welfare into veterinary and animal science education. Given the limited number of publications identified over the past 11 years, there is a clear need to promote increased interdisciplinary research, policy development, and educational reform to fully implement the One Welfare framework and align it with global sustainability goals.

## 1 Introduction

Although there is no universally accepted definition of One Health, the concept broadly emphasizes the interdependence of human, animal, and environmental health (1, 2). While the recognition of a connection—whether continuous or episodic—between human and animal health (and, at times, environmental health) has appeared throughout history, it has gained renewed prominence in recent years (2, 3). Over the past few decades, global challenges such as emerging and re-emerging zoonotic diseases, antimicrobial resistance, water scarcity, environmental pollution, food safety and insecurity, and the accelerating pace of environmental change have underlined the intricate linkages among ecosystems, animals, and human health. These developments have reinforced the relevance of the One Health framework (3). Parallel to this, the One Welfare framework has emerged, extending beyond the health-centric focus of One Health to encompass welfare. One Welfare highlights the relationship between animal welfare, human well-being, and the physical and social environment (1, 4). It is intended to foster interdisciplinary collaboration on welfare-related issues by recognizing their shared foundations and interconnected outcomes (4).

The first publication that discussed the concept of One Welfare was a commentary entitled “*One welfare: a call to develop a broader framework of thought and action*” by Colonius and Earley (5), where the authors stated that the separation between human, environmental, and animal welfare to be an artificial compartmentalization, as all these disciplines relied on the same set of scientific measures and strongly depended on each other in an ecological context. Substantial work has been undertaken over the past decade to articulate a coherent global definition and conceptual framework for One Welfare. Pioneering efforts by García-Pinillos (6) laid the groundwork for a structured understanding of One Welfare as an integrative paradigm, explicitly bridging the domains of animal welfare, human well-being, and environmental integrity. These early frameworks were instrumental in shifting the conversation from a predominantly sectorial approach to one that recognizes the multi-directional relationships among welfare dimensions. Further elaboration by Garcia-Pinillos (4) emphasized not only the theoretical underpinnings but also the practical mechanisms for implementation—particularly through inter-professional collaboration, policy inclusion, and systems thinking. The framework identifies several priority areas where a One Welfare approach can produce meaningful cross-sectoral benefits (4). These include the prevention of both animal and human abuse, improved social cohesion through shared concern for animal welfare, and the role of animal welfare in poverty alleviation and community resilience, particularly in low-income rural settings. Additionally, enhancements in animal welfare have been shown to intersect positively with food safety, farming productivity, farmer mental health, and broader issues of food security and sustainability (4). This systems-based perspective acknowledges that improvements in one domain often catalyze gains in others, reinforcing the interconnectedness that lies at the heart of the One Welfare model (6). Over time, the academic landscape has reflected growing interest in these interdisciplinary linkages. There is a clear upward trend in studies that integrate human psychosocial metrics, environmental variables, and animal welfare indicators within the same research design (7–9). For instance, King et al. (10) exemplified this integrative approach through their investigation of dairy farm systems, where they assessed cow welfare, milk production, and product quality alongside farmer well-being, using validated psychometric tools to measure stress, resilience, anxiety, and depression. A similar trend is observed in publications examining the relationship between animal welfare and potential human and/or environmental benefits. For instance, in their comprehensive review of the scientific literature on cat temperament, de Castro Travnik et al. (11) delve into the broader implications of feline temperament for human–animal matching, the quality of the human–animal bond, and the welfare of both the cat and the caregiver. Such discussions reflect growing awareness of the multifaceted impacts of animal welfare beyond the animal itself. However, with a few notable exceptions (e.g., 12), even recent studies proposing animal welfare assessment protocols frequently neglect to incorporate explicit measures of human well-being or environmental sustainability—such as biodiversity conservation—within the structure of the protocols themselves.

This study aims to present the first bibliometric review of publications that deeply engage with the One Welfare concept from February 2013—marking the publication of the foundational commentary by Colonius and Earley—through December 2024. Specifically, the study attempts to fill the lack of quantitative analysis on how the field of One Welfare has evolved over time, identifying underexplored areas and emerging themes, and evaluates the extent to which the core dimensions of One Welfare such as the integration of human, animal, and environmental welfare, are embedded within the existing body of work.

## 2 Materials and methods

### 2.1 Literature search

The aim of the current study was to quantitatively analyze publication trends and thematic developments within the One Welfare literature across time periods. Therefore, a bibliometric review was the most suitable approach for this purpose, as it allows for mapping research trends and identifying knowledge gaps that might not be as easily detected through narrative, scoping, or systematic reviews. The earliest publication identified that explicitly discusses the concept of One Welfare is the 2013 commentary by Colonius and Earley. Therefore, the literature search started from the year 2013 till December 2024. A comprehensive literature search was conducted across multiple electronic databases, including Google Scholar, PubMed, Elsevier, Springer, Web of Science, Scopus, and CABI, as well as the One Welfare website,^1^ which contained several papers discussing One Welfare topics. The search strategy employed a wide range of key terms used as topics or subject headings, which were translated into the world’s most widely spoken languages—Hindi, Chinese, Spanish, English, and French—by the authors of this paper, with assistance from native Chinese and Hindi speakers (for the full list of search terms, refer to Supplementary Table S1). The inclusion criteria for literature selection were as follows: full peer-reviewed text articles published in journals; dissertations; books or book chapters; conference proceedings; and articles from websites (that discussed in detailed the One welfare concept). Publications were considered without geographical restrictions that addressed the interconnected aspects of animal, environmental, and human welfare within the One Welfare framework. From an initial pool of 212 publications, only those that explicitly engaged with the One Welfare concept—rather than merely mentioning it incidentally, such as only in the keywords or in a single sentence within or at the end of the paper (e.g., “the current study could be included in the One Welfare framework”)—were retained. The initial literature search was performed by author SP using the platforms described above to generate a preliminary list of potentially relevant publications. This list was then shared with author SN. Both authors (SP and SN) independently and blindly screened the records according to predefined inclusion and exclusion criteria to determine their eligibility. Following the independent screening, the authors compared their selections and resolved any discrepancies through discussion, reaching consensus on the final set of publications included in the study. A total of 111 publications met the final selection criteria by providing substantive discussion on One Welfare across various disciplines (Supplementary Table S2).

### 2.2 Data extraction and analysis

Each publication was classified into three broad categories based on publication type: BOOK - full, and chapter; FULL TEXT - journal, dissertation, and website; and CONFERENCE PROCEEDINGS. This classification was determined solely by publication type, and did not consider whether the content was scientific or intended for a lay audience. Additionally, publications were categorized into four primary categories based on four main topics of policies, veterinary sciences, resource management, and human animal bond:

1. *Policy, Governance, Economy (PGE)*. This category includes studies that analyze the role of governance, public policy, and legal structures in shaping One Welfare initiatives, as well as the economic implications of implementing One Welfare approaches. Additionally, it incorporates educational and philosophical perspectives on One Welfare, including public awareness and ethics.
2. *Applied Human Animal Sciences (AHAS)*. This category includes scientific and technical research that applies One Welfare principles within veterinary science, epidemiology, and animal management, with an emphasis on animal welfare, and disease control.
3. *Societal, Economic, and Environmental Dimensions (SEED)*. This category explores the intersection of One Welfare with social structures, and environmental sustainability. It highlights how traditional knowledge, community engagement, and resource management contribute to One Welfare outcomes.
4. *Human Animal Bond and Mental health (HAB-MH)*. This category examines how relationships with animals impact mental health, therapy, and community well-being within the One Welfare framework.

Each of the four primary categories was further divided into two subcategories based on specific subtopics (Table 1). The inclusion of items within each primary category and subcategory was based on a thorough analysis of the content discussed in each publication. Because the content of each item was not confined to a single subject, the primary categories and subcategories were not considered mutually exclusive within each publication (item). Therefore, each item could include more than one primary category and subcategory. In addition, each publication was further characterized by: (1) animal type—wild animals (WA), companion animals (CA), working/sport animals (WS), production animals (PA), or general (GE, denoting non-species-specific or multi-species discussions); (2) number of papers each year; (3) world distribution of publications depending on the first author affiliation; (4) temporal context: First Time Period (FTP) (Yrs 2013–2018), and Second Time Period (STP) (Yrs 2019–2024); (5) language used in the publication (search within the five main spoken languages: Hindi, Chinese, Spanish, English, French, and Arabic); (6) inclusion of the term *One Welfare* in the main title; and (7) number of time each item was cited. This multidimensional classification enabled quantitative analysis of thematic, and methodological trends across the One Welfare literature.

**Table 1:** List of the primary categories and description of the subcategories used for the literature classification.

N.	Primary Category	Subcategory	Subcategory description
1	Policy, Governance, Economy (PGE)	LFE -Legal Frameworks and Economy	This subcategory includes research on the development and implementation of One Welfare policies, legal frameworks, economic models, and international treaties that shape welfare-oriented governance.
		EP - Education, Philosophy	This subcategory includes research on public awareness, educational programs, and ethical views within the One Welfare framework.
2	Societal, Economic, and Environmental Dimensions (SEED)	SRM - Sustainable Resource Management	This subcategory includes studies on integrating One Welfare into environmental conservation, climate action, and sustainable land use.
		TKSI - Traditional Knowledge and Societal Impacts	This subcategory includes studies on how cultural heritage wisdom in sustainable resources usage align with One Welfare principles, and it also examines the social implications of One Welfare, including food security, and rural development.
3	Applied Human Animal Sciences (AHAS)	AM - Animal Management	This subcategory focuses on research related to the care, welfare, and management of animals across various sectors, including livestock farming, wildlife conservation, laboratory animal care, and companion animal welfare. It includes studies on breeding practices, housing conditions, and nutrition that influence animal well-being.
		HAD - Human Animal Diseases	This subcategory explores the interactions between human and animal health, emphasizing zoonotic disease prevention, epidemiology, and One Health applications. It includes research on disease surveillance, biosecurity measures, antimicrobial resistance, and the role of wildlife in emerging infectious diseases
4	Human Animal Bond and Mental health (HAB-MH)	HAI - Human Animal Interaction	This subcategory examines the various ways humans and animals interact, and the broader social, emotional, and behavioral effects of these relationships. It includes research on animal-assisted interventions, working animals, pet ownership, and community-based programs that leverage human-animal connections to enhance well-being.
		PSY - Psychology	This subcategory focuses on the psychological effects of human-animal relationships, including their role in mental health, emotional resilience, and overall well-being. It includes studies on how interactions with animals reduce stress, anxiety, and depression, as well as animal-human abuse.

### 2.3 Statistical analysis

Statistical analysis was performed using R 4.4.1 (13). Chi-square goodness of fit tests were conducted to test if the number of publications differed from an equal distribution among the different categories, subcategories, studied species, and country of affiliation of the first author. Chi-square tests of independence were also used to test statistical relationships between the species, category, subcategory, and time periods. Where overall significant associations were found (*p* < 0.05), Pearson’s standardized residuals was used to identify specific cell-level deviations that contributed most to the Chi-square significance. In addition, a logistic regression using the ‘glm’ function from the ‘stats’ package was fitted using a ‘quasipoisson’ family to investigate the link between citation and number species, country, and subcategory. Due to a high degree of correlation between the primary category and subcategory, it was not possible to assess the association between citation count and primary category. A wald chi-square test was used to extract *p* values from the model and pairwise comparisons were conducted using appropriate data subsetting and a Bonferroni correction.

## 3 Results

A total of 111 publications were selected from the literature review and classified based on primary categories and subcategories. Among the selected literature, the number of items has shown a steady increase over the 11-year period considered, starting from the first publication on One Welfare in 2013 till 2024 (Table 2). In addition, the global distribution of publications based on the first authors’ country of affiliation was significantly different (χ2 = 527.14, df = 21, *p* < 0.001), with the highest number of items originated from the UK, followed by the USA and Australia (Table 3). Most of the items (97) were written in English as main language, with 10 items in Spanish, 2 items in French, and 2 items in dual-language such as English-Arabic and English-French. Among the 111 items, 66 reported the word *One Welfare* in the main title.

**Table 2:** Total number of publications for each year.

Year	Publications
2013	1
2014	1
2015	2
2016	1
2017	4
2018	9
2019	13
2020	13
2021	17
2022	20
2023	18
2024	12

**Table 3:** Worldwide distribution of publications by country based on the first authors’ affiliations.

Country	Publications
Argentina	1
Australia	15
Belgium	3
Brazil	3
Canada	8
Chile	2
China	1
Colombia	6
Denmark	1
France	7
Germany	1
Italy	7
Mexico	3
Netherlands	1
Norway	1
New Zealand	2
Romania	1
South Africa	1
Spain	1
Sweden	3
UK	28
USA	16

**Figure 1:**
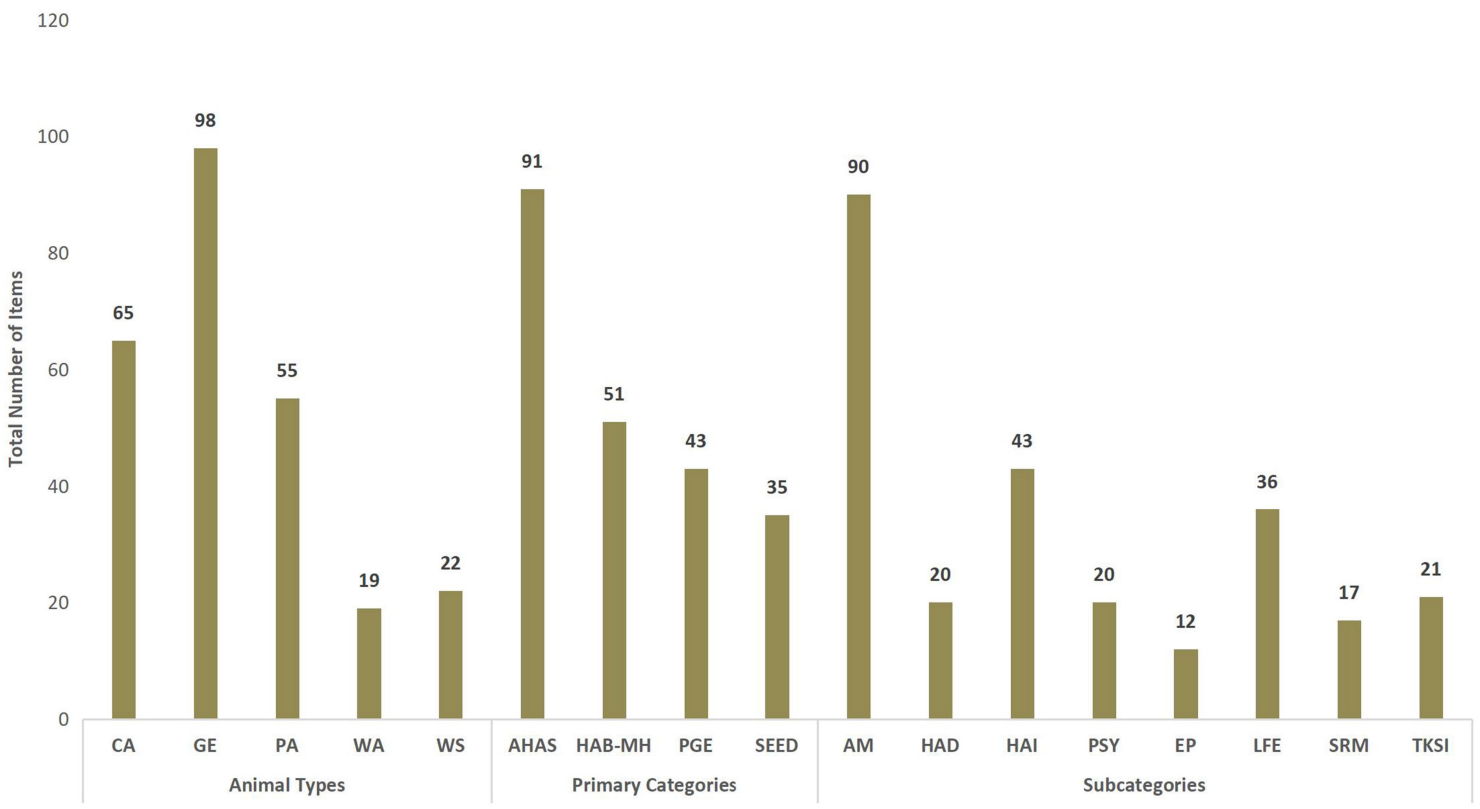
Total number of publications across categories, and subcategories, and animal type. Counts reflect non-mutually exclusive classifications, as individual publications may be assigned to multiple primary categories and subcategories. Therefore, summed counts for both primary categories and subcategories exceed the total number of publications (*n* = 111). Primary categories are not mutually exclusive within publications; thus, their total count does not reflect the total publication count. Similarly, subcategories do not reflect the total publication count nor the total number of primary categories, as a single publication may include multiple subcategories within the same primary category. The total count of *animal types* also does not correspond to the total number of publications, but rather relates to the total number of subcategories. *Animal Types* - PA: Production Animals; WA: Wild Animals; WS: Working and Sport Animals; CA: Companion Animals; GE: General. *Primary Categories* - PGE: Policy-Governance-economic; AHAS: Animal Health-Applied sciences; SEED: Societal-Economic-Environmental Dimensions; HAB-MH: Human Animal Bond & Mental health. *Subcategories* - LFE: Legal Framework & Economy; EP: Education-Philosophy; AM: Animal Management; HAD: Human Animal Diseases; TKSI: Traditional Knowledge-Societal Impacts; SRM: Sustainable Resource Management; HAI: Human Animal Interaction; Psy: Psychology.

Overall, the total number of publications was significantly different depending on the category (χ2 = 46.63, df = 3, *p* < 0.001), subcategory (χ2 = 138.19, df = 7, p < 0.001), and animal types (χ2 = 82.78, df = 4, *p* < 0.001). Among the primary categories, AHAS showed the highest number of related publications followed by the HAB-MH, PGE, and SEED categories. Among the subcategories, AM (Animal Management) accounted for the highest number of related publications, followed by HAI (Human-Animal Interactions) and LFE (Legal Framework and Economy), while the remaining subcategories demonstrated comparatively lower publication outputs. Among the animal types, general (GE) was present in most of the items, followed by companion animals (CA), production animals (PA), working/sport animals (WS), with wild animals (WA) showing the lowest number of related publications (Figure 1).

**Figure 2:**
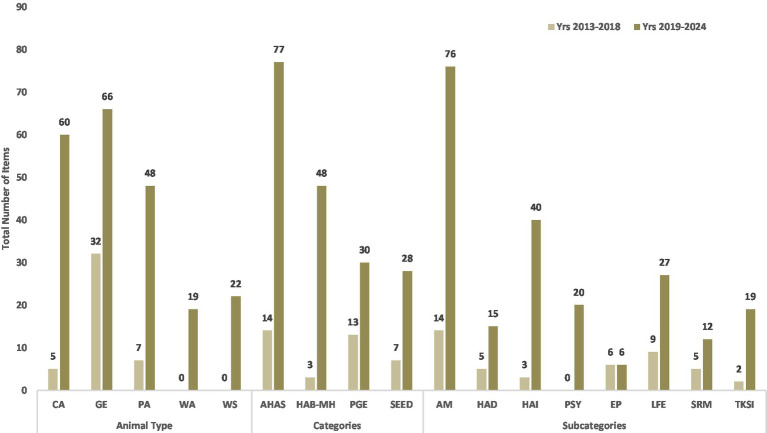
Total number of publications across categories, and subcategories, and animal type during the two time periods considered (Yrs 2013–2018 and Yrs 2019–2024). Counts reflect non-mutually exclusive classifications since individual publications may be assigned to multiple primary categories and subcategories. In addition, animal type might be included in more than one item. Thus, summed counts for both primary categories, subcategories, and animal type might exceed the total publication count (111). *Animal Types* - CA: companion animals; GE: general; PA: production animals; WA: wild animals; WS: working/sport animals. *Primary Categories* - AHAS: Applied Human Animal Sciences; PGE: Policy, Governance, Economy; SEED: Socio-Economic-Environmental Dimensions; HAB-MH: Human Animal Bond & Mental Health. *Subcategories* - LFE: Legal Framework & Economy; EP: Education & Philosophy; AM: Animal Management; HAD: Human Animal Diseases; TKSI: Traditional Knowledge and Societal Impacts; SRM: Sustainable Resource Management; HAI: Human Animal Interaction; PSY: Psychology.

Thematic shifts between the two time periods considered (Yrs 2013–2018; Yrs 2019–2024) were recorded within the target animal type, primary categories, and subcategories, with publication showing a significant higher number during Second Time Period (STP) than in First Time Period (FTP) (χ2 = 112.9, df = 1, *p* < 0.001), with one notable exception of the Education and Philosophy (EP) subcategory, which did not show changes across the two periods (Figure 2).

**Figure 3:**
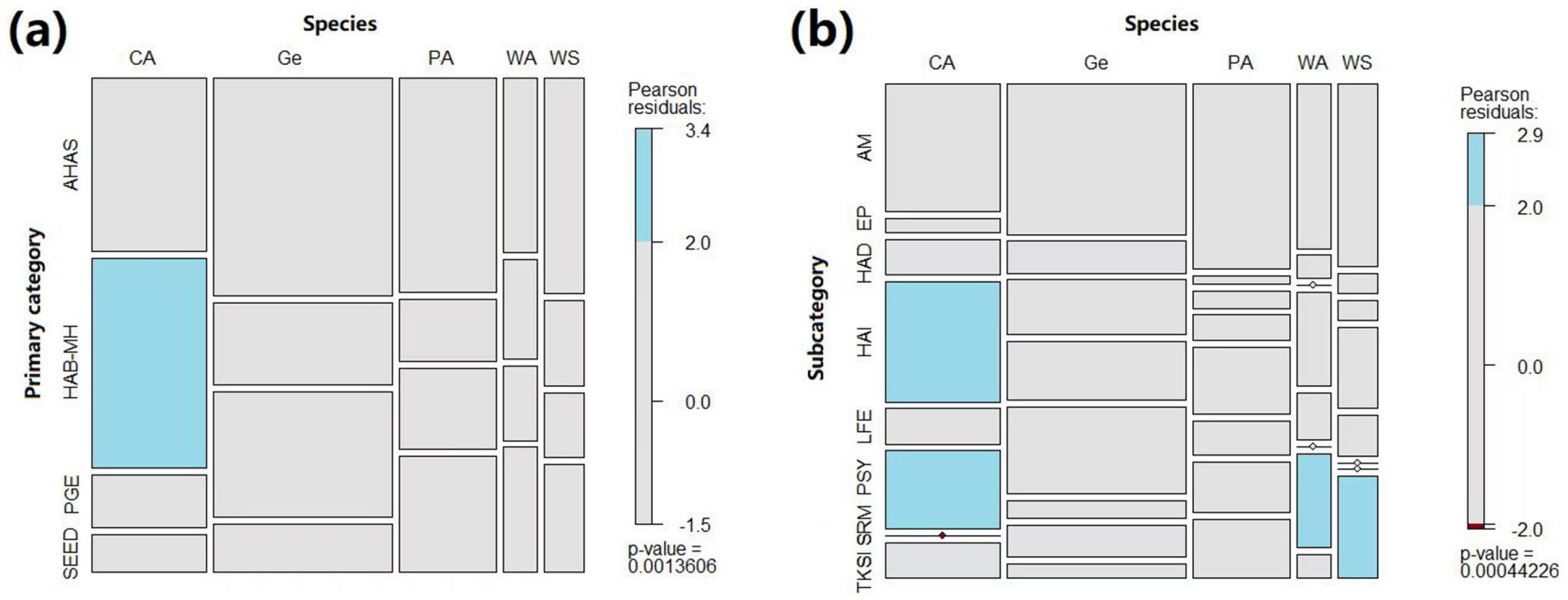
Associations between the animal type and the publication primary category **(a)** and subcategory **(b)**. Colored cases indicate frequencies that are statistically higher (red) or lower (blue) than the expected frequencies if the variables were independent. *Primary Categories*—PGE: Policy-Governance-economic; AHAS, Animal Health-Applied sciences; SEED, Societal-Economic-Environmental Dimensions; HAB-MH, Human Animal Bond and Mental health. *Subcategories*—LFE, Legal Framework and Economy; EP, Education-Philosophy; AM, Animal Management; HAD, Human Animal Diseases; TKSI, Traditional Knowledge-Societal Impacts; SRM, Sustainable Resource Management; HAI, Human Animal Interaction; Psy, Psychology. *Animal Types*—PA, Production Animals; WA, Wild Animals; WS, Working and Sport Animals; CA, Companion Animals; GE, General.

Furthermore, the category (χ2 = 32.05, df = 12, *p* = 0.001) and subcategory (χ2 = 59.72, df = 28, *p* < 0.001) were significantly linked with the target animal types, with more companion animal (CA) publications found in the HAB-MH category and HAI and PSY subcategories, but less in the SRM subcategory, while more wild animal (WA) publications in the SRM subcategory, and more working/sport animal (WS) publications in the TKSI subcategory than expected with independent variables (Figure 3).

**Figure 4:**
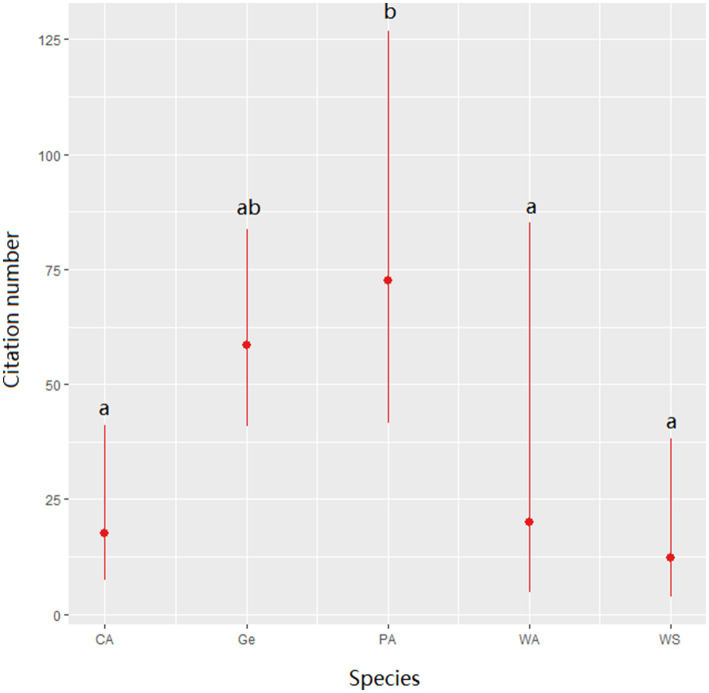
Number of citations of a publication depending on the species of interest. CA: Companion Animals; Ge: General; PA: Production Animals; WA: Wild Animals; WS: Working and Sport Animals. Different letters denote a significant difference in citation number (Wald-chisquare test with Bonferroni correction).

The number of citations was significantly impacted by the animal types (χ2 = 20.60, df = 4, *p* = 0.0004) with significantly more citations for PA species (Figure 4). The country (χ2 = 27.08, df = 21, *p* = 0.168), and subcategory (χ2 = 6.46, df = 4, *p* = 0.487) did not significantly impact the citation number.

## 4 Discussion

The concept of One Welfare, which emphasizes the interconnectedness of animal, human, and environmental well-being, has gained significant attention in recent years (14). However, the current bibliometric analysis identified only 111 publications addressing this paradigm over an 11-year period (2013–2024), suggesting it remains a nascent yet evolving field. Specifically, only 18 publications were produced during the first six-year period (2013–2018), whereas many publications emerged in the second period (2019–2024). One possible explanation for this marked increase is the publication of the One Welfare framework by García Pinillos (14), which may have heightened scientific interest in this area. In addition, more publications originated from English-speaking countries, with the highest number from the UK, followed by the USA and Australia. A similar trend was reported in a previous study on publication patterns in the field of animal welfare, which also identified the UK, Australia, and the USA as the leading contributors (15). One possible explanation for this pattern is that these countries—especially the UK—have a long and well-established tradition in animal welfare study and research (16). Furthermore, the COVID-19 pandemic may have also contributed to the surge in publications by highlighting critical gaps in zoonotic risk mitigation, human–animal–environment interactions, and socio-environmental resilience, thereby encouraging further research in these fields (17). Parallel drivers, such as escalating climate crises and global sustainability imperatives, may further explain this trajectory (18, 19).

The Applied Human-Animal Science (AHAS) primary category, with its Animal Management (AM) subcategory, emerged as the most prevalent themes across the analyzed literature. This trend was mostly evident during the Second Time Period (STP), reflecting the growing interest and importance of this themes in both academic and practical contexts (20–22). The AM subcategory, which includes publications related to animal welfare and husbandry, represents a growing subject, with publication output specifically addressing animal welfare showing an increase of 10–15% annually (23–25). Moreover, within AM, production animals (PA) emerged as a central research focus, ranking as the third most prevalent animal type across the overall selected literature. This trend was further highlighted by the association between the number of citations and animal type, with production animals receiving the highest number of citations. In general, livestock farming is critical for food security and employment, particularly in developing countries where it supports a broader societal and cultural aim then merely food production (20, 26, 27). The economic significance of production animals coupled with the sector’s rapid growth, and its global intensification, highlights the need for stakeholders to prioritize animal welfare improvements in relation to human welfare (28). In fact, studies have consistently shown that good animal husbandry practices not only enhance animal welfare but also contribute to the well-being of human communities relying on livestock (27, 29–32). Furthermore, within the Applied Human-Animal Science (AHAS) primary category, the Human-Animal Diseases (HAD) subcategory - while not among the most prevalent topics in the analysis - revealed crucial human-animal health interconnections. For example, relevant publications highlighted (1) the importance of biosecurity, specifically in relation to COVID19 pandemic (33), and (2) risks associated with carcass disposal and zoonotic transmission in intensive production systems (33). These findings underline the importance of integrating human, animal, and environmental health into policy and practice, as advocated by the One Welfare framework (33).

Companion animals (CA) emerged as the second most represented animal group in the current analysis, highlighting their sustained importance within the welfare discourse, and reflecting their integral role in human lives, both as family members, and as a critical component of animal-assisted interventions (34–38). In addition, CA were most frequently associated with the Human–Animal Bond–Mental Health (HAB–MH) primary category and its Human–Animal Interaction (HAI) subcategory, both of which exhibited a significant increase during the second time period considered, ranking as the second most prevalent themes in the overall literature. A prominent theme within the Human-Animal Interaction (HAI) subcategory involved companion animal management during emergencies. Research demonstrates that while owners directly influence their pets’ welfare, systemic factors often lie beyond individual control (39–41). For example, the recent COVID19 pandemic particularly elucidates this challenge, revealing critical gaps in emergency planning for companion animals (41). In addition, corresponding studies also highlighted effective community-based solutions, including low-cost veterinary services, mobile clinics, and foster programs to support the owners and their pets during the moments of crises (42–44).

Furthermore, companion animals-related items were also the most represented animal species within the Psychology (PSY) subcategory recognizing the great influence that pets exercise on human social dynamics (45), improving mental health, and supporting therapeutic interventions in diverse health care contexts, from hospitals and nursing homes to rehabilitation facilities (36, 37, 46). Numerous studies demonstrated how animal-assisted interventions (AAIs) improve human well-being by reducing anxiety and depression, especially during crises (47, 48). A key theme in the PSY subcategory involves the well-documented association between animal abuse and interpersonal violence, reinforcing the connection between human and animal welfare (39, 49). While less explored, similar mental health connections emerged in production animal contexts, particularly regarding (1) psychological challenges among livestock workers, and (2) correlations between animal and human abuse in agricultural settings (10, 50, 51). Despite being highly represented in the literature analysis, companion animal studies were among the least cited animal types. This could be because, although companion animal research is frequently published due to its accessibility and societal relevance, it might have received less attention as a result of its fragmented and niche topics, and lower policy relevance (23).

The General (GE) animal category—which includes publications not focused on a specific animal type or those generalized to all animal groups—was the most represented animal category across all items, in both time periods considered, and the second most cited among the animal types. This result might reflect a predominant research focus on policy development, economic implications, and conceptual alignment of One Welfare with broader global discussions about human-animal-environment interconnectedness (7). In fact, these publications frequently emphasized integrating One Welfare with One Health and One Biology frameworks to advance interdisciplinary collaborations (5, 16, 19, 52–55). Furthermore, while GE-related publications remained predominant in the current analysis, the second period (2019–2024) saw the emergence of studies focusing on specific animal types. Precisely, working/sport animals (WS) and wild animals (WA) - entirely absent during the first period (2013–2018) - emerged as a new research focus during the 2019–2024 time period. Specifically, for WS, this shift could reflect the growing recognition of this animal class’s critical roles in supporting rural and indigenous communities (56–58). This is further evidenced by the strong association between WS and the Traditional Knowledge and Societal Impacts (TKSI) subcategory, which falls under the Societal, Economic, and Environmental Dimensions (SEED) primary category, the least represented among the selected literature. For example, emerging research highlighted the vital socioeconomic contributions specifically of working animals, particularly in marginalized communities (59), revealing that (1) working animals serve as crucial income sources, especially for women who are often primary caretakers, (2) women frequently lack access to veterinary care and welfare education for working animals (horses, mules, donkeys), and (3) despite their importance, working animals remain overlooked in livestock policies, leaving them vulnerable to low welfare standards (59, 60). Their overlooked status is further reflected in their position as the least cited animal type within the literature analyzed. Addressing this gap requires targeted policy interventions and recognition of the gendered dimensions of animal care.

Similarly, although still less prominent compared to other animal-related themes, publications focused on wild animals also emerged in the second time period, and were predominantly associated with the Sustainable Resource Management (SRM) subcategory. The growing interest in wild animals in the literature was also evident from the fact that they were the third most cited among the animal types. Research addressing climate change impacts on ecosystem services, biodiversity, and sustainable production systems—central to the SRM subcategory within the Societal, Economic, and Environmental Dimensions (SEED) primary category—demonstrated modest growth during this period. However, studies related to SRM, as well as those focusing on wild animals, remained underrepresented even in the 2019–2024 time period. These studies highlighted critical issues such as the impacts of habitat loss, climate change, and human activities on wild animal populations (61–67). They also discussed the limitations of traditional conservation approaches, which often prioritize short-term benefits at the expense of long-term sustainability (64). Moreover, papers within the SRM subcategory emphasized the urgent need for holistic strategies to address interconnected challenges such as climate change, food security, and human health (68). For instance, the impact of climate change on livestock production—including aspects such as nutrition, housing, and welfare—was noted as a significant threat to productivity and economic stability (69). Sustainable agricultural models that integrate ecological solutions were proposed as key to mitigating these effects and promoting resilience, particularly among small-scale farmers (70). Several SRM-related publications were also closely linked to the Legal Framework and Economy (LFE) subcategory, underlining the importance of robust environmental welfare policies in supporting both animal and human well-being (1, 68, 70).

The primary category Policy, Governance, and Economy (PGE) was the second most represented theme within the selected literature during the first time period and the third most represented during the second time period, with the Legal Framework and Economy (LFE) subcategory serving as the dominant thematic focus. The LFE theme focused on the broader ethical, economic, and legal implications of animal welfare (4, 5, 9, 27, 71–73). For example, while animal welfare science has the potential to shape global discussions on sustainability and food security, it still remains underrepresented in major international legal and sustainability frameworks, such as the Sustainable Development Goals (SDGs) (75). Initiatives like the proposed United Nations Convention on Animal Health and Protection (UNCAHP) seek to address this gap by advocating for the establishment of global animal welfare standards (74, 75). Additionally, European Union agencies have adopted several conventions and regulations that establish specific welfare requirements for animals within the livestock industry (73). Therefore, the integration of animal welfare considerations into sustainability frameworks would not only enhance their overall effectiveness, but also better reflect the interconnectedness of human, animal, and environmental well-being (4).

As previously mentioned, the Education and Philosophy (EP) subcategory was significantly underrepresented in the reviewed literature, during both time periods considered, with only a few studies addressing the importance of enhancing knowledge about One Welfare (76–79). Currently, veterinary and animal sciences curricula disproportionately focus on animals, limiting professionals’ ability to address the broader impacts of animal care on human well-being and environmental sustainability (77). For instance, One Welfare practices have shown how “the well-being of both animals and owners are intertwined,” emphasizing the importance of a balanced approach (76). By expanding the veterinary and animal science curriculum to include a stronger emphasis on human and environmental sectors, future professionals will be better equipped to apply the One Welfare approach in practical, real-world scenarios (78). This shift is vital to ensure they can address complex challenges that require a holistic perspective, fostering a deeper integration of animal welfare, human well-being, and environmental health (4). Moreover, it would also be beneficial to integrate One Welfare principles into broader university curricula beyond veterinary medicine—such as public health, environmental sciences, and social sciences, thereby fostering a truly interdisciplinary understanding of One Welfare and its applications across different professional fields.

## 5 Conclusion

This bibliometric analysis of One Welfare publications from 2013 to 2024 reveals a growing acknowledgment of the interdependence among animal, human, and environmental well-being, as framed by the One Welfare concept (19). Interest in this paradigm increased during the second time period considered, which also encompassed the pandemic period. This upward trend in publications may reflect a growing recognition of the urgent need for holistic approaches to manage zoonotic risks, strengthen human-animal relationships, and address broader socio-environmental challenges (17). Yet, with only 111 publications identified over 11 years, One Welfare remains an emerging field demanding increased attention and interdisciplinary collaborations. AHAS and AM-related themes dominate the literature (23, 24), particularly studies focused on production animals (PA) due to their role in food security and economic resilience, especially in low-income regions (20, 26, 29, 31). Human-animal interaction (HAI) research has also gained increased attention, especially concerning companion animals (CA) and their role in supporting mental health during crises (11, 37, 46). However, critical gaps persist. Research on wild animals (WA), climate change, and sustainable resource management (SRM) remains limited (56, 61). Likewise, working animals (WS), essential to the livelihoods of marginalized groups, particularly women, are underrepresented in policy discourse (59, 60). Moreover, the integration of animal welfare into global frameworks like the SDGs remains insufficient (6, 75). Education is another undervalued domain, necessitating the inclusion of One Welfare principles in veterinary curricula (76, 78).

While the reviewed literature demonstrated a continuous grow of interest in the One Welfare field, there is still a clear need for more comprehensive research, policy development, and educational initiatives to bridge existing gaps. By fostering collaboration across animal sciences, social sciences, and environmental disciplines, the One Welfare approach can serve as a transformative framework to promote the interconnected well-being of humans, animals, and ecosystems (4, 18, 67).

## Footnotes

^1^ https://www.onewelfareworld.org/

## References

[1] Cox A. What is one health? Definitional struggles and implications for public health. Public Health Perspect. (2022) 34:110–8.

[2] Lindenmayer D. Integrating health and ecology in the Anthropocene. EcoHealth. (2022) 19:56–64.

[3] Evans B Leighton F. A history of one health. Rev Sci Tech. (2014) 33:413–20. doi: 10.20506/rst.33.2.2298, PMID: 25707172

[4] Pinillos RG. One welfare: a framework to improve animal welfare and human well-being. Vet Rec. (2018) 182:514–6.29483148

[5] Colonius T Earley R. One welfare: a call to develop a broader framework of thought and action. J Am Vet Med Assoc. (2013) 242:309–10. doi: 10.2460/javma.242.3.309, PMID: 23327170

[6] Pinillos RG. Consultation to define a One Welfare framework. Vet Rec. (2017) 180:184. doi: 10.1136/vr.j827 28213430

[7] Tarazona AM Ceballos MC Broom DM. Human relationships with domestic and other animals: one health, one welfare, one biology. Animals. (2019) 10:43. doi: 10.3390/ani10010043, PMID: 31878310 PMC7022888

[8] Broom DM. Animal welfare in relation to human welfare and sustainability–a review paper. Vet Arh. (2022) 92:541–7. doi: 10.24099/vet.arhiv.2011

[9] McBride EA Baugh S. Animal welfare in context: historical, scientific, ethical, moral and one welfare perspectives In: Human/animal relationships in transformation: Scientific, moral and legal perspectives. Eds A. Vitale, S. Pollo, Cham: Springer International Publishing (2022). 119–147.

[10] King MTM Matson RD DeVries TJ. Connecting farmer mental health with cow health and welfare on dairy farms using robotic milking systems. Anim Welf. (2021) 30:25–38. doi: 10.7120/09627286.30.1.025

[11] de Castro Travnik L Biondi V Oliveira D Serpell J. Temperament in cats: a review of its measurement and associations with welfare. Appl Anim Behav Sci. (2020) 224:104928.

[12] Leconstant C Spitz E. Integrative model of human-animal interactions: A one health -one welfare systemic approach to studying HAI. Front. Vet. Sci. (2022) 9:656833. doi: 10.3389/fvets.2022.656833 35968006 PMC9372562

[13] R Development Core Team. R: A Language and Environment for Statistical Computing. Vienna, Austria: R Foundation for Statistical Computing. (2018).

[14] Pinillos RG. One welfare: A framework to improve animal welfare and human well-being CAB International (2018). Available at: https://www.cabidigitallibrary.org/

[15] Freire R Nicol CJ. A bibliometric analysis of past and emergent trends in animal welfare science. Anim Welf. (2019) 28:465–85. doi: 10.7120/09627286.28.4.465

[16] Sinclair M Lee NY Hötzel MJ de Luna MCT Sharma A Idris M. International perceptions of animals and the importance of their welfare. Front Anim Sci. (2022) 3:960379. doi: 10.3389/fanim.2022.960379

[17] Pinillos RG. One welfare impacts of COVID-19–a summary of key highlights within the one welfare framework. Appl Anim Behav Sci. (2021) 236:105262. doi: 10.1016/j.applanim.2021.105262, PMID: 33612900 PMC7885704

[18] Alfaro AA Cortés ME. Perception of the impact of climate change on the quality of life and well-being of the inhabitants of the Cerro Blanco agricultural community, Limarí Province, Chile. Idesia. (2020) 38:127–31. doi: 10.4067/S0718-34292020000400127

[19] Pinillos RG Appleby MC Manteca X Scott-Park F Smith C Velarde A. One welfare–a platform for improving human and animal welfare. Vet Rec. (2016) 179:412–3. doi: 10.1136/vr.i5470, PMID: 27770094

[20] Rodenburg J Büchi L Haggar J. Adoption by adaptation: moving from conservation agriculture to conservation practices. Int J Agric Sustain. (2021) 19:437–55. doi: 10.1080/14735903.2020.1785734

[21] Pinillos RG. One welfare – a complement to one health supporting a sustainable dairy industry approach. In International project outcome, IDF animal health report (2018) 12, 15–16.

[22] Pinillos RG. One welfare, companion animals and their vets. Companion Anim. (2018) 23:598–8. doi: 10.12968/coan.2018.23.10.598

[23] Walker M Diez-Leon M Mason G. Animal welfare science: recent publication trends and future research priorities. Int J Comp Psychol. (2014) 27:80–100. doi: 10.46867/ijcp.2014.27.01.03

[24] Mellor DJ Beausoleil NJ Littlewood KE McLean AN McGreevy PD Jones B. The 2020 five domains model: including human–animal interactions in assessments of animal welfare. Animals. (2020) 10:1870. doi: 10.3390/ani10101870, PMID: 33066335 PMC7602120

[25] Walker MD Duggan G Roulston N Van Slack A Mason G. Negative affective states and their effects on morbidity, mortality and longevity. Anim Welf. (2012) 21:497–509. doi: 10.7120/09627286.21.4.497

[26] Herrero M Grace D Njuki J Johnson N Enahoro D Silvestri S. The roles of livestock in developing countries. Animal. (2013) 7:3–18. doi: 10.1017/S1751731112001954 23121696

[27] Pinillos R.G. (2018) One health and one welfare for all. CABI blog. Available online at: https://blog.cabi.org/2018/04/30/on-world-health-day-one-health-and-one-welfare-for-all-by-rebeca-garcia-pinilos/ (Accessed March 31, 2023)

[28] Sinclair M Fryer C Phillips CJ. The benefits of improving animal welfare from the perspective of livestock stakeholders across Asia. Animals. (2019) 9:123. doi: 10.3390/ani9040123, PMID: 30925747 PMC6524158

[29] McGlone JJ. Farm animal welfare in the context of other society issues: toward sustainable systems. Livest Prod Sci. (2001) 72:75–81. doi: 10.1016/S0301-6226(01)00268-8

[30] de Passillé AM Rushen J. Can we measure human–animal interactions in on-farm animal welfare assessment?: some unresolved issues. Appl Anim Behav Sci. (2005) 92:193–209. doi: 10.1016/j.applanim.2005.05.006

[31] Chen M Weary DM. Cattle welfare is basically human welfare: workers' perceptions of ‘animal welfare' on two dairies in China. Front Vet Sci. (2022) 8:808767. doi: 10.3389/fvets.2021.808767, PMID: 35211535 PMC8861200

[32] Collin A Bonnefous C Leterrier C Tallet C Merlot E Montagne L. One welfare for farm animals and humans: practitioners’ and citizens’ expectations. In: Improving Sustainability and welfare in Organic Poultry and Pig Production. Joint Meeting of the OK Net-EcoFeed, PPILOW, FreeBirds and POWER projects. (2021) Available at: https://ok-net-ecofeed.eu/wp-content/uploads/2021/03/1_2_One-Welfare-for-farm-animals-and-humans-practitioners-and-citizens_Collin_Niemi.pdf (Accessed 25 January, 2021).

[33] Marchant-Forde JN Boyle LA. COVID-19 effects on livestock production: a one welfare issue. Front Vet Sci. (2020) 7:585787. doi: 10.3389/fvets.2020.585787, PMID: 33195613 PMC7554581

[34] Hoy-Gerlach J Townsend L. Reimagining healthcare: human–animal bond support as a primary, secondary, and tertiary public health intervention. Int J Environ Res Public Health. (2023) 20:5272. doi: 10.3390/ijerph20075272, PMID: 37047888 PMC10094350

[35] Ly LH Gordon E Protopopova A. Inequitable flow of animals in and out of shelters: comparison of community-level vulnerability for owner-surrendered and subsequently adopted animals. Front Vet Sci. (2021) 8:784389. doi: 10.3389/fvets.2021.784389, PMID: 34869751 PMC8635993

[36] McDowall S Hazel SJ Chittleborough C Hamilton-Bruce A Stuckey R Howell TJ. The impact of the social determinants of human health on companion animal welfare. Animals. (2023) 13:1113.34. doi: 10.3390/ani13061113, PMID: 36978653 PMC10044303

[37] Travnik IDC Machado DDS Gonçalves LDS Ceballos MC Sant’Anna AC. Temperament in domestic cats: a review of proximate mechanisms, methods of assessment, its effects on human—cat relationships, and one welfare. Animals. (2020) 10:1516. doi: 10.3390/ani10091516, PMID: 32867072 PMC7552130

[38] Rodriguez KE Guérin NA Gabriels RL Serpell JA Schreiner PJ O’haire ME. The state of assessment in human-animal interaction research. Hum Anim Interact Bull. (2018) 6:63–81. doi: 10.1079/hai.2018.0022

[39] Mota-Rojas D Monsalve S Lezama-García K Mora-Medina P Domínguez-Oliva A Ramírez-Necoechea R. Animal abuse as an indicator of domestic violence: one health, one welfare approach. Animals. (2022) 12:977. doi: 10.3390/ani12080977, PMID: 35454224 PMC9024712

[40] Naud J. Incorporating shelter dogs into an animal assisted therapy program: One welfare approach [Master of Science Thesis]. Michigan State University (2022), 1–24.

[41] Squance H MacDonald C Stewart C Prasanna R Johnston DM. Strategies for implementing a one welfare framework into emergency management. Animals. (2021) 11:3141. doi: 10.3390/ani11113141, PMID: 34827873 PMC8614288

[42] Ly LH Gordon E Protopopova A. Exploring the relationship between human social deprivation and animal surrender to shelters in British Columbia, Canada. Front Vet Sci. (2021) 8:213. doi: 10.3389/fvets.2021.656597 PMC8006318 33791357

[43] Weiss E Slater M Garrison L Drain N Dolan E Scarlett JM. Large dog relinquishment to two municipal facilities in new York City and Washington, DC: identifying targets for intervention. Animals. (2014) 4:409–33. doi: 10.3390/ani4030409, PMID: 26480315 PMC4494313

[44] White B Yeung P Chilvers BL O’Donoghue K. Reducing the “cost of caring” in animal-care professionals: social work contribution in a pilot education program to address burnout and compassion fatigue. J Hum Behav Soc Environ. (2021) 31:828–47. doi: 10.1080/10911359.2020.1822249

[45] Broom DM Johnson KG. Stress and animal welfare: Key issues in the biology of humans and other animals. In: DM Broom and KG Johnson, editors. One welfare, one health, one stress: Humans and other animals. Cham: Springer Press (2019), 1–13.

[46] Johnson A Eccles E. Animal welfare considerations in animal-assisted interventions. Human Anim Inter Bull. (2022) 10:99–105. doi: 10.1079/hai.2022.0001

[47] Pirrone F. Animal assisted intervention (AAI) for children in either research, practice or policy from a one health perspective. Ann Ist Super Sanita. (2017) 53:273–4. doi: 10.4415/ANN170401 29297855

[48] Headey B Grabka MM. Pets and human health in Germany and Australia: national longitudinal results. Soc Indic Res. (2007) 80:297–311. doi: 10.1007/s11205-005-5072-z

[49] Monsalve S Hammerschmidt J Ribeiro M Caleme MVD Marconcin S Filius G. A one welfare approach to identify socioeconomic vulnerability in families during investigations into companion animal abuse in Pinhais, Brazil. Anim Welf. (2023) 32:e27. doi: 10.1017/awf.2023.18, PMID: 38487421 PMC10936322

[50] Spigarelli C Berton M Corazzin M Gallo L Pinterits S Ramanzin M. Animal welfare and farmers' satisfaction in small-scale dairy farms in the eastern Alps: a “one welfare” approach. Front Vet Sci. (2021) 8:741497. doi: 10.3389/fvets.2021.741497, PMID: 34859086 PMC8631494

[51] One Welfare Phoenix Advisory Board (2020) One welfare Phoenix – Supporting the dairy industry to recognise the interconnections between animal and human abuse and neglect. In “International project outcome”, IDF animal health report 14, 34.

[52] Fawcett A. Is a one welfare approach the key to addressing unintended harms and maximising benefits associated with animal shelters? J Appl Anim Ethics Res. (2019) 1:177–208. doi: 10.1163/25889567-12340010

[53] Lerner H. A proposal for a comprehensive human–animal approach of evaluation for animal-assisted interventions. Int J Environ Res Public Health. (2019) 16:4305. doi: 10.3390/ijerph16224305, PMID: 31698680 PMC6888089

[54] Lloyd Janice (2019) A one health/one welfare approach to dog ownership in rural and remote indigenous communities. In: Proceedings of the One Welfare Conference II 14–15 October 2019, Sydney, NSW, Australia, 44–47.

[55] Pinillos GR. One welfare’: a framework to support the implementation of OIE animal welfare standards. Bull OIE. (2017) 1:3–8. doi: 10.20506/bull.2017.1.2588

[56] Haddy E Rodrigues JB Raw Z Burden F Proops L. Documenting the welfare and role of working equids in rural communities of Portugal and Spain. Animals. (2020) 10:790. doi: 10.3390/ani10050790, PMID: 32370244 PMC7277599

[57] Vasanthakumar MA Upjohn MM Watson TL Dwyer CM. ‘All my animals are equal, but none can survive without the horse’. The contribution of working equids to the livelihoods of women across six communities in the Chimaltenango region of Guatemala. Animals. (2021) 11:1509. doi: 10.3390/ani11061509, PMID: 34067461 PMC8224632

[58] Rodrigues JB Raw Z Santurtun E Cooke F Clancy C. Donkeys in transition: changing use in a changing world. Braz J Vet Res Anim Sci. (2021) 58:e174325–5. doi: 10.11606/issn.1678-4456.bjvras.2021.174325

[59] Valette D. Invisible helpers. Women's views on the contributions of working donkeys, horses and mules to their lives. CABI Digital Library. (2014) 34:47.

[60] Kristjanson P Waters-Bayer A Johnson N Tipilda A Njuki J Baltenweck I. Livestock and women’s livelihoods. In: Quisumbing AR Meinzen-Dick R Raney TL Croppenstedt A Behrman JA, editors. Gender in agriculture: Closing the knowledge gap. Amber Peterman: Springer (2014). 209–33.

[61] Kennedy BP Boyle N Fleming PJ Harvey AM Jones B Ramp D. Ethical treatment of invasive and native fauna in Australia: perspectives through the one welfare lens. Animals. (2022) 12:1405. doi: 10.3390/ani12111405, PMID: 35681870 PMC9179540

[62] Fawcett A. One welfare, the role of health professionals, and climate change. Anim Sent. (2020) 5:9. doi: 10.51291/2377-7478.1636, PMID: 40000996

[63] Jones B Herbert C Finnerty S Kennedy B Lykins A Martin JM. In situ provisioning wildlife with food, water, or shelter after bushfires: using a one welfare framework to guide responses. Animals. (2023) 13:3518. doi: 10.3390/ani13223518, PMID: 38003136 PMC10668798

[64] Van de Wat A Garaï ME Burnett MM Henley MD Di Minin E Streicher JP. Integrating a “one well-being” approach in elephant conservation: evaluating consequences of management interventions. Ecol Soc. (2024) 29:15. doi: 10.5751/ES-15193-290315

[65] Collins MK Magle SB Gallo T. Global trends in urban wildlife ecology and conservation. Biol Conserv. (2021) 261:109236. doi: 10.1016/j.biocon.2021.109236

[66] Flint M Flint J. Use of soybean as an alternative protein source for welfare-orientated production of American alligators (*Alligator mississippiensis*). PeerJ. (2023) 11:e16321. doi: 10.7717/peerj.16321, PMID: 37904841 PMC10613434

[67] Broom DM. One biology, sustainable and regenerative farming: a role for pig and poultry production? In: D'Silva J McKenna C, editors. Regenerative farming and sustainable diets. London: Routledge (2024). 107–15.

[68] Buller H Blokhuis H Jensen P Keeling L. Towards farm animal welfare and sustainability. Animals. (2018) 8:81. doi: 10.3390/ani8060081, PMID: 29799456 PMC6025272

[69] McShane K. Why animal welfare is not biodiversity, ecosystem services, or human welfare: toward a more complete assessment of climate impacts. Les Ateliers Léthique. (2018) 13:43–64. doi: 10.7202/1055117ar

[70] Lamonaca E Bouzid A Caroprese M Ciliberti MG Cordovil CM Karatzia MA. A framework towards resilient Mediterranean eco-solutions for small-scale farming systems. Agric Food Secur. (2022) 11:1–9. doi: 10.1186/s40066-022-00399-w

[71] Sayers J Forrest R. Te Ao Maori and one welfare in Aotearoa New Zealand: the case of Kur, dog registration, the law, and local councils In: Tallberg L Hamilton L, editors. The Oxford handbook of animal organization studies. Oxford: Oxford Handbooks (2022). 425–41. doi: 10.1093/oxfordhb/9780192848185.013.28

[72] Verniers E. One health, one welfare, one right: introducing animal rights in Europe. J Europ Environ Plan Law. (2022) 19:277–310. doi: 10.1163/18760104-19040002

[73] Mirabito L. European and international projects for animal welfare: towards one welfare? In: Bulletin des GTV, (Numero Special) (2015). 21–8. doi: 10.5555/20153431796

[74] Verniers E. (2020). COVID-19 as impetus for an integrative approach to global animal welfare law. In Young legal researchers conference (YLRC). Available online at: http://hdl.handle.net/1854/LU-8685219 (Accessed February 15, 2025).

[75] Verniers E. Bringing animal welfare under the umbrella of sustainable development: a legal analysis. Rev Eur Comp Int Environ Law. (2021) 30:349–62. doi: 10.1111/reel.12414

[76] Jordan T Lem M. One health, one welfare: education in practice veterinary students’ experiences with community veterinary outreach. Can Vet J. (2014) 55:1203–6. PMID: 25477552 PMC4231813

[77] Fawcett A Mullan S McGreevy P. Application of Fraser’s “practical” ethic in veterinary practice, and its compatibility with a “one welfare” framework. Animals. (2018) 8:109. doi: 10.3390/ani8070109, PMID: 29970832 PMC6071015

[78] McGreevy PD Fawcett A Johnson J Freire R Collins T Degeling C. Review of the online one welfare portal: shared curriculum resources for veterinary undergraduate learning and teaching in animal welfare and ethics. Animals. (2020) 10:1341. doi: 10.3390/ani10081341, PMID: 32756492 PMC7460400

[79] Mota-Rojas D Orihuela A Strappini-Asteggiano A Cajiao-Pachón MN Agüera-Buendía E Mora-Medina P. Teaching animal welfare in veterinary schools in Latin America. Int J Vet Sci Med. (2018) 6:131–40. doi: 10.1016/j.ijvsm.2018.07.003, PMID: 30564587 PMC6286393

