# Supplementary material for: One welfare: bibliometric review of world literature
Source: Front Vet Sci. 2025 Aug 25;12:1627981. doi: 10.3389/fvets.2025.1627981 (PMC12427028; doi:10.3389/fvets.2025.1627981)
Supplement: Supplementary file 1 [file Data_Sheet_1.PDF]

Table S1. Full list of key terms used for the literature search

English	Hindi	Chinese	Spanish	French
**1-** *one welfare*<br>**2-** *one-welfare*<br>**3-** *one welfare in farm animals*<br>**4-** *one welfare in pet animals*<br>**5-** *one welfare in wild animals*<br>**6-** *one welfare in laboratory animals*<br>**7-** *one welfare in companion animals*<br>**8-** *one welfare in service animals*<br>**9-** *one welfare in working animals*<br>**10-** *one welfare and wildlife*<br>**11-** *one welfare and companion animals*<br>**12-** *one welfare and pet animals*<br>**13-** *one welfare and laboratory animals*<br>**14-** *one welfare and service animals*<br>**15-** *one welfare and farm animals*<br>**16-** one welfare and sustainability<br>**17-** *one welfare and dogs*<br>**18-** *one welfare and cats*<br>**19-** *one welfare and cattle*<br>**20-** *one welfare and pigs*<br>**21-** *one welfare and exotic animals*<br>**22-** *one welfare and ruminants*<br>**23-** *one welfare and goats*<br>**24-** *one welfare and horses*<br>**25-** *one welfare and animal abuse*<br>**26-** *one welfare and family abuse*<br>**27-** *one welfare and climate change*<br>**28-** *“one welfare” and “animal assisted intervention”*<br>**29-** *“one welfare” and “service dog”*<br>**30-** *“one welfare” and “assistance dog”*	**1.** एक सलामती<br>**2.** खेत जानवरों में एक सलामती<br>**3.** पालतू पशुओं में एक सलामती<br>**4.** जंगली जानवरों में एक सलामती<br>**5.** प्रयोगशाला पशुओं में एक सलामती<br>**6.** साथी जानवरों में एक सलामती<br>**7.** सेवा पशुओं में एक सलामती<br>**8.** काम करने वाले जानवरों में एक सलामती<br>**9.** एक सलामती और वन्य जीवन<br>**10.** एक सलामती और साथी जानवर<br>**11.** एक सलामती और पालतू जानवर<br>**12.** एक सलामती और प्रयोगशाला पशु<br>**13.** एक सलामती और सेवा पशु<br>**14.** एक सलामती और खेत जानवर<br>**15.** एक सलामती और वहनीयता<br>**16.** एक सलामती और कुत्ते<br>**17.** एक सलामती और बिल्लियाँ<br>**18.** एक सलामती और पशु<br>**19.** एक सलामती और सुअर<br>**20.** एक सलामती और विदेशी जानवर<br>**21.** एक सलामती और जुगाली करने वाले पशुओं<br>**22.** एक सलामती और बकरियों<br>**23.** एक सलामती और घोड़ों<br>**24.** एक सलामती और जानवरो के साथ दुर्व्यवहार	**1-** *单一福利*<br>**2-** *农场动物的单一福利*<br>**3-** *宠物动物的单一福利*<br>**4-** *野生动物的单一福利*<br>**5-** *实验动物的单一福利*<br>**6-** *伴侣动物的单一福利*<br>**7-** *服务动物的单一福利*<br>**8-** *工作动物的单一福利*<br>**9-** *单一福利与野生动物*<br>**10-** *单一福利与伴侣动物*<br>**11-** *单一福利与宠物动物*<br>**12-** *单一福利与实验动物*<br>**13-** *单一福利与服务动物*<br>**14-** *单一福利与农场动物*<br>**15-** *单一福利与可持续性*<br>**16-** *单一福利与狗*<br>**17-** *单一福利与猫*<br>**18-** *单一福利与牛*<br>**19-** *单一福利与猪*<br>**20-** *单一福利与外来动物*<br>**21-** *单一福利与反刍动物*<br>**22-** *单一福利与山羊*<br>**23-** *单一福利与马*<br>**24-** *单一福利与动物虐待*<br>**25-** *单一福利与家庭虐待*<br>**26-** *单一福利与气候变化*<br>**27-** *“单一福利”与“动物辅助干*	**1-** *bienestar único*<br>**2-** *bienestar-único*<br>**3-** *bienestar único en animales de granja*<br>**4-** *bienestar único en animales de compañía*<br>**5-** *bienestar único en animales selvaje*<br>**6-** *bienestar único en animales de laboratorio*<br>**7-** *bienestar único en animales de compañía*<br>**8-** *bienestar único en animales de servicio*<br>**9-** *bienestar único en animales de trabajo*<br>**10-** *bienestar único y fauna silvestre*<br>**11-** *bienestar único y animales de compañía*<br>**12-** *bienestar único y animales de compañía*<br>**13-** *bienestar único y animales de laboratorio*<br>**14-** *bienestar único y animales de servicio*<br>**15-** *bienestar único y animales de granja*<br>**16-** *bienestar único y sostenibilidad*<br>**17-** *bienestar único y perros*	**1-** *bien-être unique*<br>**2-** *bien-être unique chez les animaux d’élevage*<br>**3-** *bien-être unique chez les animaux de compagnie*<br>**4-** *bien-être unique chez les animaux sauvages*<br>**5-** *bien-être unique chez les animaux de laboratoire*<br>**6-** *bien-être unique animaux de compagnie*<br>**7-** *bien-être unique animaux d’assistance*<br>**8-** *bien-être unique animaux de travail*<br>**9-** *bien-être unique et faune sauvage*<br>**10-** *bien-être unique et animaux de compagnie*<br>**11-** *bien-être unique et animaux de compagnie*<br>**12-** *bien-être unique et animaux de laboratoire*<br>**13-** *bien-être unique et animaux d’assistance*<br>**14-** *bien-être unique et animaux d’élevage*<br>**15-** *bien-être unique et durabilité*<br>**16-** *bien-être unique et chiens*<br>**17-** *bien-être unique et chats*

**31-** *“one welfare” and “service animal”*<br>**32-** *“one welfare” and “assistance animal”*	**25.** एक सलामती और पारिवारिक दुर्व्यवहार<br>**26.** एक सलामती और जलवायु परिवर्तन<br>**27.** एक सलामती और पशु सहायता हस्तक्षेप<br>**28.** एक सलामती और सेवा कुत्ता<br>**29.** एक सलामती और सहायता कुत्ता<br>**30.** एक सलामती और सेवा पशु<br>**31.** एक सलामती और सहायता पशु	*预”*<br>**28-** *“单一福利”与“服务犬”*<br>**29-** *“单一福利”与“辅助犬”*<br>**30-** *“单一福利”与“服务动物”*<br>**31-** *“单一福利”与“辅助动物”*	**18-** *bienestar único y gatos*<br>**19-** *bienestar único y ganado bovino*<br>**20-** *bienestar único y cerdos*<br>**21-** *bienestar único y animales exóticos*<br>**22-** *bienestar único y rumiantes*<br>**23-** *bienestar único y cabras*<br>**24-** *bienestar único y caballos*<br>**25-** *bienestar único y violencia en animales*<br>**26-** *bienestar único y violencia familiar*<br>**27-** *bienestar único y cambio climático*<br>**28-** *“bienestar único” y “intervención asistida con animales”*<br>**29-** *“bienestar único” y “perro de servicio”*<br>**30-** *“bienestar único” y “perro de asistencia”*<br>**31-** *“bienestar único” y “animal de servicio”*<br>**32-** *“bienestar único” y “animal de asistencia”*	**18-** *bien-être unique et bovins*<br>**19-** *bien-être unique et porcs*<br>**20-** *bien-être unique et animaux exotiques*<br>**21-** *bien-être unique et ruminants*<br>**22-** *bien-être unique et chèvres*<br>**23-** *bien-être unique et chevaux*<br>**24-** *bien-être unique et maltraitance animale*<br>**25-** *bien-être unique et violence familiale*<br>**26-** *bien-être unique et changement climatique*<br>**27-** *« bien-être unique » et « intervention assistée par l’animal »*<br>**28-** *« bien-être unique » et « chien de service »*<br>**29-** *« bien-être unique » et « chien d’assistance »*<br>**30-** *« bien-être unique » et « animal de service »*<br>**31-** *« bien-être unique » et « animal d’assistance »*

Table S2. List of the 109 publication classified depending on *animal types* (CA: companion animals; GE: general; PR: production animals; WA: wild animals; WS: working/sport animals), *primary categories* (AHAS- Applied Human Animal Sciences; PGE - Policy, Governance, Economy; SEED - Socio-Economic-Environmental Dimensions; HAB-MH - Human Animal Bond & Mental Health), *subcategories* (LFE - Legal Framework & Economy; EP - Education & Philosophy; AM- Animal Management; HAD - Human Animal Diseases; TKSI - Traditional Knowledge and Societal Impacts; SRM - Sustainable Resource Management; HAI - Human Animal Interaction; PSY - Psychology), *Year Group* (First Time Period: FTP; Second Time Period: STP), *One Welfare in the Main Title* (Yes; No), and *Language*.

N.	Year Pub	Year Group	Pub.Type	First Author Origin	N. of Citations	Primary category	Subcategory	Animal species	One Welfare in Main title	Language	Citation
1	2021	STP	Full Text (Journal)	UK	46	AHAS	AM	Ge	Yes	English	Pinillos, R. G. (2021). One welfare impacts of COVID-19–A summary of key highlights within the one welfare framework. *Applied Animal Behaviour Science*, *236*, 105262.
1	2021	STP	Full Text (Journal)	UK	46	HAB-MH	HAI	Ge	Yes	English	Pinillos, R. G. (2021). One welfare impacts of COVID-19–A summary of key highlights within the one welfare framework. *Applied Animal Behaviour Science*, *236*, 105262.
1	2021	STP	Full Text (Journal)	UK	46	AHAS	HAD	Ge	Yes	English	Pinillos, R. G. (2021). One welfare impacts of COVID-19–A summary of key highlights within the one welfare framework. Applied Animal Behaviour Science, 236, 105262.
2	2013	FTP	Full Text (Journal)	USA	101	AHAS	AM	Ge	Yes	English	Colonius, T. J., & Earley, R. W. (2013). One welfare: A call to develop a broader framework of thought and action. *Journal of the American Veterinary Medical Association*, *242*(3), 309-310.
2	2013	FTP	Full Text (Journal)	USA	101	SEED	TKSI	Ge	Yes	English	Colonius, T. J., & Earley, R. W. (2013). One welfare: A call to develop a broader framework of thought and action. *Journal of the American Veterinary Medical Association*,

											242(3), 309-310.
2	2013	FTP	Full Text (Journal)	USA	101	PGE	LPE	Ge	Yes	English	Colonius, T. J., & Earley, R. W. (2013). One welfare: A call to develop a broader framework of thought and action. *Journal of the American Veterinary Medical Association*, *242*(3), 309-310.
2	2013	FTP	Full Text (Journal)	USA	101	PGE	EP	Ge	Yes	English	Colonius, T. J., & Earley, R. W. (2013). One welfare: A call to develop a broader framework of thought and action. *Journal of the American Veterinary Medical Association*, *242*(3), 309-310.
3	2020	STP	Full Text (Journal)	Brazil	35	HAB-MH	HAI	CA	Yes	English	Travnik, I. D. C., Machado, D. D. S., Gonçalves, L. D. S., Ceballos, M. C., & Sant'Anna, A. C. (2020). Temperament in domestic cats: a review of proximate mechanisms, methods of assessment, its effects on human—cat relationships, and one welfare. *Animals*, *10*(9), 1516.
3	2020	STP	Full Text (Journal)	Brazil	35	AHAS	AM	CA	Yes	English	Travnik, I. D. C., Machado, D. D. S., Gonçalves, L. D. S., Ceballos, M. C., & Sant'Anna, A. C. (2020). Temperament in domestic cats: a review of proximate mechanisms, methods of assessment, its effects on human—cat relationships, and one welfare. *Animals*, *10*(9), 1516.
4	2019	STP	Full Text (Journal)	Italy	49	HAB-MH	HAI	WA	No	English	de Mori, B., Ferrante, L., Florio, D., Macchi, E., Pollastri, I., & Normando, S. (2019). A protocol for the ethical assessment of wild animal–visitor interactions (AVIP) evaluating animal welfare, education, and conservation

											outcomes. *Animals*, *9*(8), 487.
4	2019	STP	Full Text (Journal)	Italy	49	PGE	EP	WA	No	English	de Mori, B., Ferrante, L., Florio, D., Macchi, E., Pollastri, I., & Normando, S. (2019). A protocol for the ethical assessment of wild animal–visitor interactions (AVIP) evaluating animal welfare, education, and conservation outcomes. *Animals*, *9*(8), 487.
4	2019	STP	Full Text (Journal)	Italy	49	AHAS	AM	WA	No	English	de Mori, B., Ferrante, L., Florio, D., Macchi, E., Pollastri, I., & Normando, S. (2019). A protocol for the ethical assessment of wild animal–visitor interactions (AVIP) evaluating animal welfare, education, and conservation outcomes. *Animals*, *9*(8), 487.
5	2022	STP	Full Text (Journal)	Italy	11	HAB-MH	HAI	WA	No	English	Pollastri, I., Normando, S., Florio, D., Ferrante, L., Bandoli, F., Macchi, E., ... & de Mori, B. (2022). The Animal-Visitor Interaction Protocol (AVIP) for the assessment of Lemur catta walk-in enclosure in zoos. *Plos one*, *17*(7), e0271409.
5	2022	STP	Full Text (Journal)	Italy	11	AHAS	AM	WA	No	English	Pollastri, I., Normando, S., Florio, D., Ferrante, L., Bandoli, F., Macchi, E., ... & de Mori, B. (2022). The Animal-Visitor Interaction Protocol (AVIP) for the assessment of Lemur catta walk-in enclosure in zoos. *Plos one*, *17*(7), e0271409.
6	2022	STP	Book-chapter	NZ	6	PGE	LPE	CA	Yes	English	Sayers, Janet, and Rachel Forrest, 'Te Ao M 膩 ori and One Welfare in Aotearoa New Zealand: The Case of Kur 墨, Dog Registration, the Law, and Local Councils', in Linda Tallberg, and Lindsay Hamilton (eds), The Oxford

6	2022	STP	Book-chapter	NZ	6	SEED	TKSI	CA	Yes	English	Handbook of Animal Organization Studies, Oxford Handbooks (2022; online edn, Oxford Academic, 20 Oct. 2022), https://doi.org/10.1093/oxfordhb/9780192848185.013.28, Sayers, Janet, and Rachel Forrest, 'Te Ao M 膩 ori and One Welfare in Aotearoa New Zealand: The Case of Kur 墨, Dog Registration, the Law, and Local Councils', in Linda Tallberg, and Lindsay Hamilton (eds), The Oxford Handbook of Animal Organization Studies, Oxford Handbooks (2022; online edn, Oxford Academic, 20 Oct. 2022), https://doi.org/10.1093/oxfordhb/9780192848185.013.28,
7	2019	STP	Full Text (Journal)	Colombia	0	AHAS	AM	PR	Yes	Spanish	Peñuela T.J.A. (2019). The “One Welfare” approach as a proposal within the implementation of the Sustainable Development Goals. REVISTA COLOMBIANA DE ZOOTECNIA Vol 5 (10): 11-18
7	2019	STP	Full Text (Journal)	Colombia	0	SEED	SRM	PR	Yes	Spanish	Peñuela T.J.A. (2019). The “One Welfare” approach as a proposal within the implementation of the Sustainable Development Goals. REVISTA COLOMBIANA DE ZOOTECNIA Vol 5 (10): 11-18
8	2017	FTP	Full Text (Journal)	Italy	5	HAB-MH	HAI	CA	No	English	Pirrone, F. (2017). Animal assisted intervention (AAI) for children in either research, practice or policy from a One Health

											perspective. *Annali dell'Istituto Superiore di Sanità*, *53*(4), 273-274.
8	2017	FTP	Full Text (Journal)	Italy	5	AHAS	AM	CA	No	English	Pirrone, F. (2017). Animal assisted intervention (AAI) for children in either research, practice or policy from a One Health perspective. *Annali dell'Istituto Superiore di Sanità*, *53*(4), 273-274.
8	2017	FTP	Full Text (Journal)	Italy	5	PGE	LPE	CA	No	English	Pirrone, F. (2017). Animal assisted intervention (AAI) for children in either research, practice or policy from a One Health perspective. *Annali dell'Istituto Superiore di Sanità*, *53*(4), 273-274.
9	2019	STP	Full Text (Journal)	Colombia	154	AHAS	AM	Ge	Yes	English	Tarazona, A. M., Ceballos, M. C., & Broom, D. M. (2019). Human relationships with domestic and other animals: One health, one welfare, one biology. *Animals*, *10*(1), 43.
9	2019	STP	Full Text (Journal)	Colombia	154	HAB-MH	HAI	Ge	Yes	English	Tarazona, A. M., Ceballos, M. C., & Broom, D. M. (2019). Human relationships with domestic and other animals: One health, one welfare, one biology. *Animals*, *10*(1), 43.
10	2022	STP	Full Text (Journal)	USA	1	PGE	LPE	Ge	No	English	McClellan, K. (2022). The Politics of Equivalence:(Post) Colonial Care and Transnational Animal Welfare in Jordan. *Social Anthropology/Anthropologie sociale*, *30*(3), 126-142.
10	2022	STP	Full Text (Journal)	USA	1	AHAS	AM	Ge	No	English	McClellan, K. (2022). The Politics of Equivalence:(Post) Colonial Care and Transnational Animal Welfare in Jordan. *Social Anthropology/Anthropologie sociale*, *30*(3), 126-142.

11	2022	STP	Full Text (Journal)	UK	5	AHAS	AM	Ge	No	English	Broom, D. M. (2022). Animal welfare in relation to human welfare and sustainability-a review paper.
11	2022	STP	Full Text (Journal)	UK	5	AHAS	HAD	Ge	No	English	Broom, D. M. (2022). Animal welfare in relation to human welfare and sustainability-a review paper.
11	2022	STP	Full Text (Journal)	UK	5	SEED	SRM	Ge	No	English	Broom, D. M. (2022). Animal welfare in relation to human welfare and sustainability-a review paper.
12	2018	FTP	Full Text (Journal)	USA	19	SEED	SRM	Ge	No	English	McShane, K. (2018). Why animal welfare is not biodiversity, ecosystem services, or human welfare: Toward a more complete assessment of climate impacts. *Les ateliers de l'éthique*, *13*(1), 43-64.
12	2018	FTP	Full Text (Journal)	USA	19	AHAS	AM	Ge	No	English	McShane, K. (2018). Why animal welfare is not biodiversity, ecosystem services, or human welfare: Toward a more complete assessment of climate impacts. *Les ateliers de l'éthique*, *13*(1), 43-64.
12	2018	FTP	Full Text (Journal)	USA	19	PGE	LPE	Ge	No	English	McShane, K. (2018). Why animal welfare is not biodiversity, ecosystem services, or human welfare: Toward a more complete assessment of climate impacts. *Les ateliers de l'éthique*, *13*(1), 43-64.
13	2022	STP	Full Text (Journal)	Italy	10	PGE	LPE	PR	No	English	Lamonaca, E., Bouzid, A., Caroprese, M., Ciliberti, M. G., Cordovil, C. M., Karatzia, M. A., ... & Santeramo, F. G. (2022). A framework towards resilient Mediterranean eco-solutions for small-scale farming systems. *Agriculture & Food Security*, *11*(1), 1-9.

13	2022	STP	Full Text (Journal)	Italy	10	SEED	SRM	PR	No	English	Lamonaca, E., Bouzid, A., Caroprese, M., Ciliberti, M. G., Cordovil, C. M., Karatzia, M. A., ... & Santeramo, F. G. (2022). A framework towards resilient Mediterranean eco-solutions for small-scale farming systems. *Agriculture & Food Security*, *11*(1), 1-9.
14	2019	STP	Conferen ce Proceedi ngs	Australia	0	HAB-MH	HAI	CA	Yes	English	Lloyd, Janice (2019) A One Health/One Welfare approach to dog ownership in rural and remote Indigenous communities. In: Proceedings of the One Welfare Conference II. 15. pp. 44-47. From: One Welfare Conference II, 14-15 October 2019, Sydney, NSW, Australia.
14	2019	STP	Conferen ce Proceedi ngs	Australia	0	AHAS	AM	CA	Yes	English	Lloyd, Janice (2019) A One Health/One Welfare approach to dog ownership in rural and remote Indigenous communities. In: Proceedings of the One Welfare Conference II. 15. pp. 44-47. From: One Welfare Conference II, 14-15 October 2019, Sydney, NSW, Australia.
15	2019	STP	Full Text (Journal)	Sweden	25	HAB-MH	PSY	Ge	No	English	Lerner, H. (2019). A proposal for a comprehensive human–animal approach of evaluation for animal-assisted interventions. *International Journal of Environmental Research and Public Health*, *16*(22), 4305.
15	2019	STP	Full Text (Journal)	Sweden	25	HAB-MH	HAI	Ge	No	English	Lerner, H. (2019). A proposal for a comprehensive human–animal approach of evaluation for animal-assisted interventions. *International Journal of Environmental Research and Public Health*, *16*(22), 4305.

16	2022	STP	Full Text (Journal)	Belgium	3	PGE	LPE	Ge	Yes	English	Verniers, E. (2022). One Health, One Welfare, One Right: Introducing Animal Rights in Europe. *Journal for European Environmental & Planning Law*, *19*(4), 277-310.
17	2020	STP	Full Text (Journal)	UK	3	AHAS	AM	Ge	Yes	Spanish	Pinillos, R. G. (2020). Introducción al marco One Welfare (un solo bienestar) en el contexto de la producción animal veterinaria. *Albéitar: publicación veterinaria independiente*, (239), 6-8.
18	2022	STP	Full Text (Journal)	USA	4	HAB-MH	HAI	Ge	No	English	Johnson, A., & Eccles, E. (2022). Animal welfare considerations in animal-assisted interventions. *Human-Animal Interaction Bulletin*, (2022).
18	2022	STP	Full Text (Journal)	USA	4	AHAS	AM	Ge	No	English	Johnson, A., & Eccles, E. (2022). Animal welfare considerations in animal-assisted interventions. *Human-Animal Interaction Bulletin*, (2022).
19	2022	STP	Book-chapter	UK	12	AHAS	AM	Ge	Yes	English	McBride, E. A., & Baugh, S. (2022). Animal welfare in context: historical, scientific, ethical, moral and One Welfare perspectives. In *Human/Animal Relationships in Transformation: Scientific, Moral and Legal Perspectives* (pp. 119-147). Cham: Springer International Publishing.
19	2022	STP	Book-chapter	UK	12	HAB-MH	HAI	Ge	Yes	English	McBride, E. A., & Baugh, S. (2022). Animal welfare in context: historical, scientific, ethical, moral and One Welfare perspectives. In *Human/Animal Relationships in Transformation: Scientific, Moral and Legal Perspectives* (pp. 119-147). Cham: Springer

											International Publishing.
19	2022	STP	Book-chapter	UK	12	PGE	LPE	Ge	Yes	English	McBride, E. A., & Baugh, S. (2022). Animal welfare in context: historical, scientific, ethical, moral and One Welfare perspectives. In *Human/Animal Relationships in Transformation: Scientific, Moral and Legal Perspectives* (pp. 119-147). Cham: Springer International Publishing.
20	2022	STP	Full Text (Journal)	Mexico	42	HAB-MH	PSY	Ge	Yes	English	Mota-Rojas, D., Monsalve, S., Lezama-García, K., Mora-Medina, P., Domínguez-Oliva, A., & Ramírez-Necoechea, R. Animal Abuse as an Indicator of Domestic Violence: One Health, One Welfare Approach. Animals. 2022; 12 (8): 977.
20	2022	STP	Full Text (Journal)	Mexico	42	SEED	TKSI	Ge	Yes	English	Mota-Rojas, D., Monsalve, S., Lezama-García, K., Mora-Medina, P., Domínguez-Oliva, A., & Ramírez-Necoechea, R. Animal Abuse as an Indicator of Domestic Violence: One Health, One Welfare Approach. Animals. 2022; 12 (8): 977.
20	2022	STP	Full Text (Journal)	Mexico	42	HAB-MH	HAI	Ge	Yes	English	Mota-Rojas, D., Monsalve, S., Lezama-García, K., Mora-Medina, P., Domínguez-Oliva, A., & Ramírez-Necoechea, R. Animal Abuse as an Indicator of Domestic Violence: One Health, One Welfare Approach. Animals. 2022; 12 (8): 977.
20	2022	STP	Full Text (Journal)	Mexico	42	AHAS	AM	Ge	Yes	English	Mota-Rojas, D., Monsalve, S., Lezama-García, K., Mora-Medina, P., Domínguez-Oliva, A., & Ramírez-Necoechea, R. Animal Abuse as an Indicator of Domestic Violence: One Health,

											One Welfare Approach. Animals. 2022; 12 (8): 977.
21	2021	STP	Full Text (Journal)	Germany	33	PGE	LPE	Ge	No	English	Wünderlich, N. V., Mosteller, J., Beverland, M. B., Downey, H., Kraus, K., Lin, M. H., & Syrjälä, H. (2021). Animals in our lives: An interactive well-being perspective. *Journal of Macromarketing*, *41*(4), 646-662.
21	2021	STP	Full Text (Journal)	Germany	33	HAB-MH	HAI	Ge	No	English	Wünderlich, N. V., Mosteller, J., Beverland, M. B., Downey, H., Kraus, K., Lin, M. H., & Syrjälä, H. (2021). Animals in our lives: An interactive well-being perspective. *Journal of Macromarketing*, *41*(4), 646-662.
21	2021	STP	Full Text (Journal)	Germany	33	AHAS	HAD	Ge	No	English	Wünderlich, N. V., Mosteller, J., Beverland, M. B., Downey, H., Kraus, K., Lin, M. H., & Syrjälä, H. (2021). Animals in our lives: An interactive well-being perspective. *Journal of Macromarketing*, *41*(4), 646-662.
21	2021	STP	Full Text (Journal)	Germany	33	AHAS	AM	Ge	No	English	Wünderlich, N. V., Mosteller, J., Beverland, M. B., Downey, H., Kraus, K., Lin, M. H., & Syrjälä, H. (2021). Animals in our lives: An interactive well-being perspective. *Journal of Macromarketing*, *41*(4), 646-662.
22	2018	FTP	Full Text (Journal)	Australia	27	SEED	TKSI	Ge	Yes	English	Fawcett, A., Mullan, S., & McGreevy, P. (2018). Application of Fraser's "practical" ethic in veterinary practice, and its compatibility with a "one welfare" framework. *Animals*, *8*(7), 109.
22	2018	FTP	Full Text (Journal)	Australia	27	PGE	EP	Ge	Yes	English	Fawcett, A., Mullan, S., & McGreevy, P. (2018). Application of Fraser's "practical" ethic in veterinary practice, and its

											compatibility with a “one welfare” framework. *Animals*, *8*(7), 109.
23	2020	STP	Full Text (Journal)	UK	26	AHAS	AM	WS	No	English	Haddy, E., Rodrigues, J. B., Raw, Z., Burden, F., & Proops, L. (2020). Documenting the welfare and role of working equids in rural communities of Portugal and Spain. *Animals*, *10*(5), 790.
24	2020	STP	Full Text (Journal)	Australia	14	PGE	EP	Ge	Yes	English	McGreevy, P. D., Fawcett, A., Johnson, J., Freire, R., Collins, T., Degeling, C., ... & Tzioumis, V. (2020). Review of the online One Welfare portal: Shared curriculum resources for veterinary undergraduate learning and teaching in animal welfare and ethics. *Animals*, *10*(8), 1341.
24	2020	STP	Full Text (Journal)	Australia	14	PGE	LPE	Ge	Yes	English	McGreevy, P. D., Fawcett, A., Johnson, J., Freire, R., Collins, T., Degeling, C., ... & Tzioumis, V. (2020). Review of the online One Welfare portal: Shared curriculum resources for veterinary undergraduate learning and teaching in animal welfare and ethics. *Animals*, *10*(8), 1341.
25	2021	STP	Full Text (Journal)	UK	17	AHAS	AM	WS	No	English	Vasanthakumar, M. A., Upjohn, M. M., Watson, T. L., & Dwyer, C. M. (2021). ‘All My Animals Are Equal, but None Can Survive without the Horse’. The Contribution of Working Equids to the Livelihoods of Women across Six Communities in the Chimaltenango Region of Guatemala. *Animals*, *11*(6), 1509.
25	2021	STP	Full Text (Journal)	UK	17	PGE	EP	WS	No	English	Vasanthakumar, M. A., Upjohn, M. M., Watson, T. L., & Dwyer, C. M. (2021). ‘All My

											Animals Are Equal, but None Can Survive without the Horse’. The Contribution of Working Equids to the Livelihoods of Women across Six Communities in the Chimaltenango Region of Guatemala. *Animals*, *11*(6), 1509.
25	2021	STP	Full Text (Journal)	UK	17	PGE	LPE	WS	No	English	Vasanthakumar, M. A., Upjohn, M. M., Watson, T. L., & Dwyer, C. M. (2021). ‘All My Animals Are Equal, but None Can Survive without the Horse’. The Contribution of Working Equids to the Livelihoods of Women across Six Communities in the Chimaltenango Region of Guatemala. *Animals*, *11*(6), 1509.
25	2021	STP	Full Text (Journal)	UK	17	SEED	TKSI	WS	No	English	Vasanthakumar, M. A., Upjohn, M. M., Watson, T. L., & Dwyer, C. M. (2021). ‘All My Animals Are Equal, but None Can Survive without the Horse’. The Contribution of Working Equids to the Livelihoods of Women across Six Communities in the Chimaltenango Region of Guatemala. *Animals*, *11*(6), 1509.
26	2021	STP	Full Text (Journal)	China	2	PGE	LPE	Ge	No	English	Wu, K., Yu, Y., Chen, C., & Fu, Z. (2021). Is One Health a Viable Strategy in Animal Health Litigation: Evidence from Civil Lawsuits in China. *Animals*, *11*(9), 2560.
27	2021	STP	Full Text (Journal)	NZ	12	AHAS	AM	Ge	Yes	English	Squance, H., MacDonald, C., Stewart, C., Prasanna, R., & Johnston, D. M. (2021). Strategies for implementing a one welfare framework into emergency management. *Animals*, *11*(11), 3141.
27	2021	STP	Full Text (Journal)	NZ	12	PGE	LPE	Ge	Yes	English	Squance, H., MacDonald, C., Stewart, C., Prasanna, R., & Johnston, D. M. (2021).

											Strategies for implementing a one welfare framework into emergency management. *Animals*, *11*(11), 3141.
27	2021	STP	Full Text (Journal)	NZ	12	PGE	EP	Ge	Yes	English	Squance, H., MacDonald, C., Stewart, C., Prasanna, R., & Johnston, D. M. (2021). Strategies for implementing a one welfare framework into emergency management. *Animals*, *11*(11), 3141.
28	2022	STP	Full Text (Journal)	Australia	1	AHAS	AM	Ge	Yes	English	Kennedy, B. P., Boyle, N., Fleming, P. J., Harvey, A. M., Jones, B., Ramp, D., ... & McGreevy, P. D. (2022). Ethical Treatment of Invasive and Native Fauna in Australia: Perspectives through the One Welfare Lens. *Animals*, *12*(11), 1405.
28	2022	STP	Full Text (Journal)	Australia	1	PGE	EP	Ge	Yes	English	Kennedy, B. P., Boyle, N., Fleming, P. J., Harvey, A. M., Jones, B., Ramp, D., ... & McGreevy, P. D. (2022). Ethical Treatment of Invasive and Native Fauna in Australia: Perspectives through the One Welfare Lens. *Animals*, *12*(11), 1405.
28	2022	STP	Full Text (Journal)	Australia	1	PGE	LPE	Ge	Yes	English	Kennedy, B. P., Boyle, N., Fleming, P. J., Harvey, A. M., Jones, B., Ramp, D., ... & McGreevy, P. D. (2022). Ethical Treatment of Invasive and Native Fauna in Australia: Perspectives through the One Welfare Lens. *Animals*, *12*(11), 1405.
28	2022	STP	Full Text (Journal)	Australia	1	HAB-MH	HAI	Ge	Yes	English	Kennedy, B. P., Boyle, N., Fleming, P. J., Harvey, A. M., Jones, B., Ramp, D., ... & McGreevy, P. D. (2022). Ethical Treatment of Invasive and Native Fauna in Australia:

											Perspectives through the One Welfare Lens. *Animals*, *12*(11), 1405.
29	2022	STP	Full Text (Journal)	USA	18	AHAS	AM	WS	Yes	English	Stallones, L., McManus, P., & McGreevy, P. (2023). Sustainability and the Thoroughbred breeding and racing industries: An enhanced one welfare perspective. *Animals*, *13*(3), 490.
30	2022	STP	Full Text (Journal)	Italy	4	HAB-MH	PSY	Ge	Yes	English	Baragli, P., Yngvesson, J., Gentili, C., & Lanata, A. (2022). Emotions and emotional interplay within and between species: A "one welfare" perspective. *Frontiers in Veterinary Science*, *9*, 1011214.
31	2021	STP	Full Text (Journal)	Belgium	14	PGE	LPE	Ge	No	English	Verniers, E. (2021). Bringing animal welfare under the umbrella of sustainable development: A legal analysis. *Review of European, Comparative & International Environmental Law*, *30*(3), 349-362.
32	2017	FTP	Conference Proceedings	UK	45	AHAS	AM	Ge	Yes	English	Garcia Pinillos, R. (2017). One Welfare': a framework to support the implementation of OIE animal welfare standards. *Bull. OIE*, *2017*, 3-8.
33	2022	STP	Full Text (Journal)	France	21	HAB-MH	PSY	Ge	Yes	English	Leconstant, C., & Spitz, E. (2022). Integrative model of human-animal interactions: A one health–one welfare systemic approach to studying HAI. *Frontiers in Veterinary Science*, *9*, 656833.
33	2022	STP	Full Text (Journal)	France	21	HAB-MH	HAI	Ge	Yes	English	Leconstant, C., & Spitz, E. (2022). Integrative model of human-animal interactions: A one health–one welfare systemic approach to studying HAI. *Frontiers in Veterinary Science*, *9*, 656833.

34	2018	FTP	Full Text (Website)	UK	0	AHAS	AM	Ge	Yes	English	Garcia Pinillos R (2018). One Health and One Welfare for all. CABI blog https://blog.cabi.org/2018/04/30/on-world-health-day-one-health-and-one-welfare-for-all-by-rebeca-garcia-pinilos/
34	2018	FTP	Full Text (Website)	UK	0	AHAS	HAD	Ge	Yes	English	Garcia Pinillos R (2018). One Health and One Welfare for all. CABI blog https://blog.cabi.org/2018/04/30/on-world-health-day-one-health-and-one-welfare-for-all-by-rebeca-garcia-pinilos/
35	2021	STP	Full Text (Journal)	Canada	32	AHAS	AM	PR	No	English	King, M. T. M., Matson, R. D., & DeVries, T. J. (2021). Connecting farmer mental health with cow health and welfare on dairy farms using robotic milking systems. *Animal welfare*, *30*(1), 25-38.
36	2020	STP	Conferen ce Proceedi ngs	Belgium	0	PGE	LPE	Ge	No	English	Verniers, E. (2020). COVID-19 as impetus for an integrative approach to global animal welfare law. In *Young Legal Researchers Conference (YLRC)*.
37	2017	FTP	Full Text (Journal)	Canada	28	AHAS	AM	Ge	Yes	Dual Language (E, Fr)	Bourque, T. (2017). One welfare. *The Canadian Veterinary Journal*, *58*(3), 217.
37	2017	FTP	Full Text (Journal)	Canada	28	PGE	LPE	Ge	Yes	Dual Language (E, Fr)	Bourque, T. (2017). One welfare. *The Canadian Veterinary Journal*, *58*(3), 217.
38	2014	FTP	Full Text (Journal)	USA	68	PGE	EP	CA	Yes	English	Jordan, T., & Lem, M. (2014). One health, one welfare: education in practice veterinary students’ experiences with community veterinary outreach. *The Canadian Veterinary Journal*, *55*(12), 1203.

39	2020	STP	Full Text (Website)	UK	0	AHAS	AM	CA	Yes	English	Westgarth C (2020). Dog walking – One health, one welfare. https://vetfocus.royalcanin.com/en/scientific/dog-walking-one-health-one-welfare
39	2020	STP	Full Text (Website)	UK	0	HAB-MH	PSY	CA	Yes	English	Westgarth C (2020). Dog walking – One health, one welfare. https://vetfocus.royalcanin.com/en/scientific/dog-walking-one-health-one-welfare
39	2020	STP	Full Text (Website)	UK	0	HAB-MH	HAI	CA	Yes	English	Westgarth C (2020). Dog walking – One health, one welfare. https://vetfocus.royalcanin.com/en/scientific/dog-walking-one-health-one-welfare
39	2020	STP	Full Text (Website)	UK	0	AHAS	HAD	CA	Yes	English	Westgarth C (2020). Dog walking – One health, one welfare. https://vetfocus.royalcanin.com/en/scientific/dog-walking-one-health-one-welfare
40	2019	STP	Full Text (Journal)	Spain	0	AHAS	AM	CA	Yes	Spanish	Riera, S. (2019). El proyecto One welfare ofrece un marco para la mejora del bienestar animal, humano y medioambiental. *Argos: Informativo Veterinario*, (209), 6-6.
41	2015	FTP	Full Text (Journal)	France	0	PGE	LPE	Ge	Yes	English	Mirabito, L. (2015). European and international projects for animal welfare: towards one welfare?. *Bulletin des GTV*, (Numero Special), 21-28.
41	2015	FTP	Full Text (Journal)	France	0	HAB-MH	HAI	Ge	Yes	English	Mirabito, L. (2015). European and international projects for animal welfare: towards one welfare?. *Bulletin des GTV*, (Numero Special), 21-28.
41	2015	FTP	Full Text (Journal)	France	0	AHAS	AM	Ge	Yes	English	Mirabito, L. (2015). European and international projects for animal welfare:

											towards one welfare?. *Bulletin des GTV*, (Numero Special), 21-28.
42	2021	STP	Full Text (Journal)	Canada	46	HAB-MH	PSY	CA	No	English	Ly, L. H., Gordon, E., & Protopopova, A. (2021). Exploring the relationship between human social deprivation and animal surrender to shelters in British Columbia, Canada. *Frontiers in veterinary science*, *8*, 656597.
42	2021	STP	Full Text (Journal)	Canada	46	HAB-MH	HAI	CA	No	English	Ly, L. H., Gordon, E., & Protopopova, A. (2021). Exploring the relationship between human social deprivation and animal surrender to shelters in British Columbia, Canada. *Frontiers in veterinary science*, *8*, 656597.
43	2022	STP	Full Text (Journal)	Australia	3	PGE	LPE	CA	No	English	Newton, W., Signal, T., & Judd, J. A. (2022). Fur, Fin, and Feather: Management of Animal Interactions in Australian Residential Aged Care Facilities. *Animals*, *12*(24), 3591.
43	2022	STP	Full Text (Journal)	Australia	3	HAB-MH	HAI	CA	No	English	Newton, W., Signal, T., & Judd, J. A. (2022). Fur, Fin, and Feather: Management of Animal Interactions in Australian Residential Aged Care Facilities. *Animals*, *12*(24), 3591.
43	2022	STP	Full Text (Journal)	Australia	3	AHAS	AM	CA	No	English	Newton, W., Signal, T., & Judd, J. A. (2022). Fur, Fin, and Feather: Management of Animal Interactions in Australian Residential Aged Care Facilities. *Animals*, *12*(24), 3591.
44	2021	STP	Full Text (Journal)	Italy	15	AHAS	AM	PR	Yes	English	Spigarelli, C., Berton, M., Corazzin, M., Gallo, L., Pinterits, S., Ramanzin, M., ... & Bovolenta, S. (2021). Animal Welfare and Farmers' Satisfaction in Small-Scale Dairy Farms in the

										Eastern Alps: A “One Welfare” Approach. *Frontiers in Veterinary Science*, *8*, 741497.
45	2021	STP	Full Text (Journal)	Canada	27	HAB-MH	PSY	CA	No	English	Ly, L. H., Gordon, E., & Protopopova, A. (2021). Inequitable flow of animals in and out of shelters: comparison of community-level vulnerability for owner-surrendered and subsequently adopted animals. *Frontiers in veterinary science*, *8*, 784389.
45	2021	STP	Full Text (Journal)	Canada	27	SEED	TKSI	CA	No	English	Ly, L. H., Gordon, E., & Protopopova, A. (2021). Inequitable flow of animals in and out of shelters: comparison of community-level vulnerability for owner-surrendered and subsequently adopted animals. Frontiers in veterinary science, 8, 784389.
46	2020	STP	Full Text (Website)	UK	3	AHAS	AM	Ge	Yes	Dual Language (E,Ar)	Board, O. W. P. A. (2020). *One Welfare Phoenix—Supporting the Dairy Industry to Recognise the Interconnections Between Animal and Human Abuse and Neglect* (No. 14, p. 34). IDF Animal Health Report.
46	2020	STP	Full Text (Website)	UK	3	HAB-MH	PSY	PR	Yes	Dual Language (E,Ar)	Board, O. W. P. A. (2020). *One Welfare Phoenix—Supporting the Dairy Industry to Recognise the Interconnections Between Animal and Human Abuse and Neglect* (No. 14, p. 34). IDF Animal Health Report.
46	2020	STP	Full Text (Website)	UK	3	PGE	LPE	Ge	Yes	Dual Language (E,Ar)	Board, O. W. P. A. (2020). *One Welfare Phoenix—Supporting the Dairy Industry to Recognise the Interconnections Between Animal and Human Abuse and Neglect* (No. 14, p. 34). IDF Animal Health Report.
46	2020	STP	Full Text	UK	3	HAB-MH	HAI	PR	Yes	Dual	Board, O. W. P. A. (2020). *One Welfare*

			(Website)							Language (E,Ar)	*Phoenix—Supporting the Dairy Industry to Recognise the Interconnections Between Animal and Human Abuse and Neglect* (No. 14, p. 34). IDF Animal Health Report.
47	2018	FTP	Full Text (Website)	Sweden	0	AHAS	AM	PR	Yes	English	García Pinillos R (2018). One Welfare – a complement to One Health supporting a sustainable dairy industry approach. IDF Animal Health Report • Issue N°12
47	2018	FTP	Full Text (Website)	Sweden	0	PGE	EP	PR	Yes	English	García Pinillos R (2018). One Welfare – a complement to One Health supporting a sustainable dairy industry approach. IDF Animal Health Report • Issue N°12
47	2018	FTP	Full Text (Website)	Sweden	0	PGE	LPE	PR	Yes	English	García Pinillos R (2018). One Welfare – a complement to One Health supporting a sustainable dairy industry approach. IDF Animal Health Report • Issue N°12
47	2018	FTP	Full Text (Website)	Sweden	0	AHAS	HAD	PR	Yes	English	García Pinillos R (2018). One Welfare – a complement to One Health supporting a sustainable dairy industry approach. IDF Animal Health Report • Issue N°12
48	2022	STP	Full Text (Dissertat ion)	USA	0	AHAS	AM	CA	Yes	English	Naud, J. (2022). *Incorporating Shelter Dogs into an Animal Assisted Therapy Program: A One Welfare Approach*. Michigan State University.
48	2022	STP	Full Text (Dissertat ion)	USA	0	HAB-MH	HAI	CA	Yes	English	Naud, J. (2022). *Incorporating Shelter Dogs into an Animal Assisted Therapy Program: A One Welfare Approach*. Michigan State University.
48	2022	STP	Full Text (Dissertat	USA	0	HAB-MH	PSY	CA	Yes	English	Naud, J. (2022). *Incorporating Shelter Dogs into an Animal Assisted Therapy Program: A*

			ion)								*One Welfare Approach*. Michigan State University.
49	2019	STP	Full Text (Journal)	Australia	12	HAB-MH	PSY	CA	Yes	English	Fawcett, A. (2019). Is a One Welfare approach the key to addressing unintended harms and maximising benefits associated with animal shelters?. *Journal of applied animal ethics research*, *1*(2), 177-208.
49	2019	STP	Full Text (Journal)	Australia	12	PGE	EP	CA	Yes	English	Fawcett, A. (2019). Is a One Welfare approach the key to addressing unintended harms and maximising benefits associated with animal shelters?. *Journal of applied animal ethics research*, *1*(2), 177-208.
50	2022	STP	Full Text (Journal)	UK	11	AHAS	AM	WS	Yes	English	Kubasiewicz, L. M., Watson, T., Norris, S. L., Chamberlain, N., Nye, C., Perumal, R. K., ... & Burden, F. A. (2022). One welfare: Linking poverty, equid ownership and equid welfare in the brick kilns of India. *Animal Welfare*, *31*(4), 517-528.
50	2022	STP	Full Text (Journal)	UK	11	SEED	TKSI	WS	Yes	English	Kubasiewicz, L. M., Watson, T., Norris, S. L., Chamberlain, N., Nye, C., Perumal, R. K., ... & Burden, F. A. (2022). One welfare: Linking poverty, equid ownership and equid welfare in the brick kilns of India. *Animal Welfare*, *31*(4), 517-528.
50	2022	STP	Full Text (Journal)	UK	11	HAB-MH	HAI	WS	Yes	English	Kubasiewicz, L. M., Watson, T., Norris, S. L., Chamberlain, N., Nye, C., Perumal, R. K., ... & Burden, F. A. (2022). One welfare: Linking poverty, equid ownership and equid welfare in the brick kilns of India. *Animal Welfare*, *31*(4), 517-528.

51	2020	STP	Full Text (Journal)	USA	182	HAB-MH	PSY	PR	Yes	English	Marchant-Forde, J. N., & Boyle, L. A. (2020). COVID-19 effects on livestock production: a one welfare issue. *Frontiers in veterinary science*, *7*, 585787.
51	2020	STP	Full Text (Journal)	USA	182	AHAS	AM	PR	Yes	English	Marchant-Forde, J. N., & Boyle, L. A. (2020). COVID-19 effects on livestock production: a one welfare issue. *Frontiers in veterinary science*, *7*, 585787.
51	2020	STP	Full Text (Journal)	USA	182	AHAS	HAD	PR	Yes	English	Marchant-Forde, J. N., & Boyle, L. A. (2020). COVID-19 effects on livestock production: a one welfare issue. *Frontiers in veterinary science*, *7*, 585787.
52	2021	STP	Full Text (Journal)	UK	0	AHAS	AM	Ge	Yes	English	van Dijk, C. (2021). One Health–One Welfare: An interdependent health approach. *Stockfarm*, *11*(12), 39-39.
52	2021	STP	Full Text (Journal)	UK	0	AHAS	HAD	Ge	Yes	English	van Dijk, C. (2021). One Health–One Welfare: An interdependent health approach. *Stockfarm*, *11*(12), 39-39.
53	2019	STP	Conferen ce Proceedi ngs	USA	0	AHAS	AM	Ge	Yes	English	Bayne K. (2019). One Welfare as an Extension of the One Health Initiative: The Concept and Its Status. The 66th Annual Meeting of Japanese Association for Laboratory Animal Science
53	2019	STP	Conferen ce Proceedi ngs	USA	0	AHAS	HAD	Ge	Yes	English	Bayne K. (2019). One Welfare as an Extension of the One Health Initiative: The Concept and Its Status. The 66th Annual Meeting of Japanese Association for Laboratory Animal Science
54	2021	STP	Full Text (Website)	France	0	AHAS	AM	PR	Yes	English	Collin, A., Bonnefous, C., Leterrier, C., Tallet, C., Merlot, E., Montagne, L., ... & Niemi, J.

											(2021, January). One Welfare for farm animals and humans: practitioners' and citizens' expectations. In *Improving sustainability and welfare in organic poultry and pig production*.
54	2021	STP	Full Text (Website)	France	0	PGE	LPE	PR	Yes	English	Collin, A., Bonnefous, C., Leterrier, C., Tallet, C., Merlot, E., Montagne, L., ... & Niemi, J. (2021, January). One Welfare for farm animals and humans: practitioners' and citizens' expectations. In *Improving sustainability and welfare in organic poultry and pig production*.
54	2021	STP	Full Text (Website)	France	0	SEED	TKSI	PR	Yes	English	Collin, A., Bonnefous, C., Leterrier, C., Tallet, C., Merlot, E., Montagne, L., ... & Niemi, J. (2021, January). One Welfare for farm animals and humans: practitioners' and citizens' expectations. In *Improving sustainability and welfare in organic poultry and pig production*.
54	2021	STP	Full Text (Website)	France	0	SEED	SRM	PR	Yes	English	Collin, A., Bonnefous, C., Leterrier, C., Tallet, C., Merlot, E., Montagne, L., ... & Niemi, J. (2021, January). One Welfare for farm animals and humans: practitioners' and citizens' expectations. In *Improving sustainability and welfare in organic poultry and pig production*.
55	2021	STP	Conference Proceedings	Netherland	0	AHAS	AM	PR	Yes	English	Rodenburg, B., García Pinillos, R., García Ara, A., & Schaadt, J. (2021). One Welfare in farm animal production: animal welfare in connection to human wellbeing and

											environmental sustainability. In *Proceedings Book OneWelfare World Conference*. One Welfare CIC.
56	2020	STP	Full Text (Journal)	Australia	11	AHAS	AM	Ge	Yes	English	Fawcett, A. (2020). One Welfare, the role of health professionals, and climate change. *Animal Sentience*, *5*(30), 9.
56	2020	STP	Full Text (Journal)	Australia	11	AHAS	HAD	Ge	Yes	English	Fawcett, A. (2020). One Welfare, the role of health professionals, and climate change. *Animal Sentience*, *5*(30), 9.
56	2020	STP	Full Text (Journal)	Australia	11	SEED	SRM	Ge	Yes	English	Fawcett, A. (2020). One Welfare, the role of health professionals, and climate change. *Animal Sentience*, *5*(30), 9.
57	2018	FTP	Full Text (Journal)	UK	6	AHAS	AM	CA	Yes	English	Pinillos, R. G. (2018). One Welfare, companion animals and their vets. *Companion Animal*, *23*(10), 598-598.
58	2021	STP	Full Text (Journal)	France	27	AHAS	AM	PR	No	English	Beaujouan, J., Cromer, D., & Boivin, X. (2021). From human–animal relation practice research to the development of the livestock farmer's activity: an ergonomics–applied ethology interaction. *Animal*, *15*(12), 100395.
58	2021	STP	Full Text (Journal)	France	27	HAB-MH	HAI	PR	No	English	Beaujouan, J., Cromer, D., & Boivin, X. (2021). From human–animal relation practice research to the development of the livestock farmer's activity: an ergonomics–applied ethology interaction. *Animal*, *15*(12), 100395.
59	2023	STP	Full Text (Journal)	Brazil	1	HAB-MH	PSY	CA	Yes	English	Monsalve, S., Hammerschmidt, J., Ribeiro, M., Caleme, M. V. D., Marconcin, S., Filius, G., & Garcia, R. D. C. M. (2023). A One Welfare approach to identify socioeconomic vulnerability in families during investigations

											into companion animal abuse in Pinhais, Brazil. *Animal Welfare*, *32*, e27.
59	2023	STP	Full Text (Journal)	Brazil	1	SEED	TKSI	CA	Yes	English	Monsalve, S., Hammerschmidt, J., Ribeiro, M., Caleme, M. V. D., Marconcin, S., Filius, G., & Garcia, R. D. C. M. (2023). A One Welfare approach to identify socioeconomic vulnerability in families during investigations into companion animal abuse in Pinhais, Brazil. Animal Welfare, 32, e27.
59	2023	STP	Full Text (Journal)	Brazil	1	PGE	LPE	CA	Yes	English	Monsalve, S., Hammerschmidt, J., Ribeiro, M., Caleme, M. V. D., Marconcin, S., Filius, G., & Garcia, R. D. C. M. (2023). A One Welfare approach to identify socioeconomic vulnerability in families during investigations into companion animal abuse in Pinhais, Brazil. *Animal Welfare*, *32*, e27.
60	2020	STP	Conferen ce Proceedi ngs	UK	0	AHAS	AM	Ge	Yes	Spanish	García Pinillos g. et al (2020). Welfare, Violence and the human animal interaction - One Welfare Phoenix Project. ISAE conference.
61	2023	STP	Book	UK	0	AHAS	AM	Ge	Yes	English	Richardson C. One Welfare in Practice: The Role of the Veterinarian. Anim Welf. 2023 Feb 27;32:e23. doi: 10.1017/awf.2023.13. PMCID: PMC10936269.
62	2022	STP	Full Text (Website)	UK	2	AHAS	AM	Ge	Yes	English	Cox, J. 2022 Operationalising One Health-One Welfare. https://www.wellbeingintlstudiesrepository.org/ hw_onehealth/5/
62	2022	STP	Full Text	UK	2	PGE	LPE	Ge	Yes	English	Cox, J. 2022 Operationalising One Health-One

			(Website)							Welfare. https://www.wellbeingintlstudiesrepository.org/ hw_onehealth/5/
62	2022	STP	Full Text (Website)	UK	2	AHAS	HAD	Ge	Yes	English	Cox, J. 2022 Operationalising One Health-One Welfare. https://www.wellbeingintlstudiesrepository.org/ hw_onehealth/5/
62	2022	STP	Full Text (Website)	UK	2	SEED	SRM	Ge	Yes	English	Cox, J. 2022 Operationalising One Health-One Welfare. https://www.wellbeingintlstudiesrepository.org/ hw_onehealth/5/
63	2020	STP	Conference Proceedings	France	0	AHAS	AM	PR	No	English	Bonnefous C. et al (2020). ORGANIC PIG AND POULTRY PRODUCTION: WHAT ARE THE ANIMAL WELFARE CHALLENGES? Organic World Congress.
64	2018	FTP	Book	UK	157	AHAS	AM	Ge	Yes	English	Pinillos, R. G. (Ed.). (2018). *One welfare: A framework to improve animal welfare and human well-being*. Cabi.
64	2018	FTP	Book	UK	157	AHAS	HAD	Ge	Yes	English	Pinillos, R. G. (Ed.). (2018). One welfare: A framework to improve animal welfare and human well-being. Cabi.
64	2018	FTP	Book	UK	157	SEED	SRM	Ge	Yes	English	Pinillos, R. G. (Ed.). (2018). One welfare: A framework to improve animal welfare and human well-being. Cabi.
65	2016	FTP	Full Text (Journal)	UK	498	AHAS	AM	Ge	Yes	English	Pinillos, R. G., Appleby, M. C., Manteca, X., Scott-Park, F., Smith, C., & Velarde, A. (2016). One Welfare–a platform for improving human

											and animal welfare. *Veterinary Record*, *179*(16), 412-413.
65	2016	FTP	Full Text (Journal)	UK	498	AHAS	HAD	Ge	Yes	English	Pinillos, R. G., Appleby, M. C., Manteca, X., Scott-Park, F., Smith, C., & Velarde, A. (2016). One Welfare–a platform for improving human and animal welfare. *Veterinary Record*, *179*(16), 412-413.
65	2016	FTP	Full Text (Journal)	UK	498	SEED	SRM	Ge	Yes	English	Pinillos, R. G., Appleby, M. C., Manteca, X., Scott-Park, F., Smith, C., & Velarde, A. (2016). One Welfare–a platform for improving human and animal welfare. *Veterinary Record*, *179*(16), 412-413.
66	2020	STP	Full Text (Journal)	UK	18	AHAS	AM	WS	No	English	Rodrigues, J. B., Sullivan, R. J., Judge, A., Norris, S. L., & Burden, F. A. (2020). Quantifying poor working equid welfare in Nepalese brick kilns using a welfare assessment tool. *Veterinary Record*, *187*(11), 445-445.
67	2023	STP	Full Text (Journal)	USA	9	AHAS	HAD	CA	No	English	Hoy-Gerlach, J., & Townsend, L. (2023). Reimagining healthcare: Human–animal bond support as a primary, secondary, and tertiary public health intervention. *International Journal of Environmental Research and Public Health*, *20*(7), 5272.
68	2019	STP	Full Text (Journal)	Colombia	1	AHAS	AM	Ge	Yes	Spanish	Romero Peñuela M. (2019). The transport and slaughter processes in Colombia: approach from the One Welfare concept. Academia Colombiana de Ciencia Veterinarias Volumen 8 No. 1 : 72-88
69	2021	STP	Full Text	Argentin	0	AHAS	AM	PR	No	Spanish	Ghezzi,Marcelo D.and Sepiurka,Leonardo,

			(Journal)	a							20210427495, Spanish, Journal article, Argentina, 102, (2), Buenos Aires, Revista de Medicina Veterinaria (Buenos Aires), (22–29), Sociedad de Medicina Veterinaria, Role of the veterinarian ensuring the animal welfare and food safety.
70	2022	STP	Full Text (Journal)	Canada	9	HAB-MH	PSY	CA	Yes	English	Koralesky, K. E., Rankin, J. M., & Fraser, D. (2022). The everyday work of One Welfare in animal sheltering and protection. *Humanities and Social Sciences Communications*, *9*(1), 1-10.
71	2021	STP	Full Text (Journal)	Australia	6	AHAS	AM	WA	No	English	Stephens, T. (2021). Kangaroo management and animal welfare. *Ecological Management & Restoration*, *22*, 71-74.
72	2018	FTP	Full Text (Journal)	Mexico	74	PGE	EP	Ge	No	English	Mota-Rojas, D., Orihuela, A., Strappini-Asteggiano, A., Cajiao-Pachón, M. N., Agüera-Buendía, E., Mora-Medina, P., ... & Alonso-Spilsbury, M. (2018). Teaching animal welfare in veterinary schools in Latin America. *International journal of veterinary science and medicine*, *6*(2), 131-140.
73	2019	STP	Full Text (Journal)	Australia	84	AHAS	AM	PR	No	English	Sinclair, M., Fryer, C., & Phillips, C. J. (2019). The benefits of improving animal welfare from the perspective of livestock stakeholders across Asia. *Animals*, *9*(4), 123.
73	2019	STP	Full Text (Journal)	Australia	84	SEED	TKSI	PR	No	English	Sinclair, M., Fryer, C., & Phillips, C. J. (2019). The benefits of improving animal welfare from the perspective of livestock stakeholders across Asia. *Animals*, *9*(4), 123.
73	2019	STP	Full Text	Australia	84	HAB-MH	HAI	PR	No	English	Sinclair, M., Fryer, C., & Phillips, C. J. (2019).

			(Journal)								The benefits of improving animal welfare from the perspective of livestock stakeholders across Asia. *Animals*, *9*(4), 123.
74	2023	STP	Full Text (Journal)	Australia	37	AHAS	AM	CA	No	English	McDowall, S., Hazel, S. J., Chittleborough, C., Hamilton-Bruce, A., Stuckey, R., & Howell, T. J. (2023). The impact of the social determinants of human health on companion animal welfare. *Animals*, *13*(6), 1113.
74	2023	STP	Full Text (Journal)	Australia	37	HAB-MH	PSY	CA	No	English	McDowall, S., Hazel, S. J., Chittleborough, C., Hamilton-Bruce, A., Stuckey, R., & Howell, T. J. (2023). The impact of the social determinants of human health on companion animal welfare. *Animals*, *13*(6), 1113.
74	2023	STP	Full Text (Journal)	Australia	37	HAB-MH	HAI	CA	No	English	McDowall, S., Hazel, S. J., Chittleborough, C., Hamilton-Bruce, A., Stuckey, R., & Howell, T. J. (2023). The impact of the social determinants of human health on companion animal welfare. *Animals*, *13*(6), 1113.
74	2023	STP	Full Text (Journal)	Australia	37	AHAS	HAD	CA	No	English	McDowall, S., Hazel, S. J., Chittleborough, C., Hamilton-Bruce, A., Stuckey, R., & Howell, T. J. (2023). The impact of the social determinants of human health on companion animal welfare. *Animals*, *13*(6), 1113.
75	2022	STP	Full Text (Dissertation)	UK	3	AHAS	AM	WS	No	English	Haddy, E. C. (2022). *Understanding the role of environmental and human factors in working equid welfare* (Doctoral dissertation, University of Portsmouth).
75	2022	STP	Full Text (Dissertation)	UK	3	SEED	TKSI	WS	No	English	Haddy, E. C. (2022). *Understanding the role of environmental and human factors in working equid welfare* (Doctoral dissertation,

										University of Portsmouth).
75	2022	STP	Full Text (Dissertation)	UK	3	HAB-MH	HAI	WS	No	English	Haddy, E. C. (2022). *Understanding the role of environmental and human factors in working equid welfare* (Doctoral dissertation, University of Portsmouth).
76	2018	FTP	Full Text (Journal)	UK	215	AHAS	AM	PR	No	English	Buller, H., Blokhuis, H., Jensen, P., & Keeling, L. (2018). Towards farm animal welfare and sustainability. *Animals*, *8*(6), 81.
76	2018	FTP	Full Text (Journal)	UK	215	PGE	LPE	PR	No	English	Buller, H., Blokhuis, H., Jensen, P., & Keeling, L. (2018). Towards farm animal welfare and sustainability. *Animals*, *8*(6), 81.
76	2018	FTP	Full Text (Journal)	UK	215	SEED	SRM	PR	No	English	Buller, H., Blokhuis, H., Jensen, P., & Keeling, L. (2018). Towards farm animal welfare and sustainability. *Animals*, *8*(6), 81.
77	2019	STP	Full Text (Journal)	Mexico	0	AHAS	AM	PR	Yes	Spanish	Gabriel L.R.I (2019). STRATEGIC ALLIANCES TO ACHIEVE THE "ONE WELFARE" PRECEPT. Revista Mexicana de Agroecosistemas Vol. 6 (Suplemento 2)
77	2019	STP	Full Text (Journal)	Mexico	0	PGE	LPE	PR	Yes	Spanish	Gabriel L.R.I (2019). STRATEGIC ALLIANCES TO ACHIEVE THE "ONE WELFARE" PRECEPT. Revista Mexicana de Agroecosistemas Vol. 6 (Suplemento 2)
77	2019	STP	Full Text (Journal)	Mexico	0	SEED	TKSI	PR	Yes	Spanish	Gabriel L.R.I (2019). STRATEGIC ALLIANCES TO ACHIEVE THE "ONE WELFARE" PRECEPT. Revista Mexicana de Agroecosistemas Vol. 6 (Suplemento 2)
78	2021	STP	Full Text (Dissertation)	USA	0	HAB-MH	PSY	CA	Yes	English	Andrukonis, A. (2021). *Wellbeing in animal shelters—a one welfare approach* (Doctoral dissertation).
78	2021	STP	Full Text	USA	0	AHAS	AM	CA	Yes	English	Andrukonis, A. (2021). *Wellbeing in animal*

			(Dissertation)								*shelters—a one welfare approach* (Doctoral dissertation).
78	2021	STP	Full Text (Dissertation)	USA	0	HAB-MH	HAI	CA	Yes	English	Andrukonis, A. (2021). *Wellbeing in animal shelters—a one welfare approach* (Doctoral dissertation).
79	2019	STP	Conference Proceedings	Canada	0	AHAS	AM	Ge	No	English	Fraser D. (2019). What Do We Mean by “One Welfare”? World Small Animal Veterinary Association Congress Proceedings.
79	2019	STP	Conference Proceedings	Canada	0	HAB-MH	HAI	Ge	No	English	Fraser D. (2019). What Do We Mean by “One Welfare”? World Small Animal Veterinary Association Congress Proceedings.
80	2018	FTP	Full Text (Journal)	Australia	3	PGE	EP	Ge	No	English	Fawcett, Anne and McGreevy, Paul (2018) What sets us apart could be our salvation. Animal Sentience 23(14)
81	2015	FTP	Full Text (Journal)	UK	19	PGE	LPE	Ge	Yes	English	Pinillos, R.G., Appleby, M.C., Scott-Park, F. and Smith, C.W. (2015), One Welfare. Veterinary Record, 177: 629-630. https://doi.org/10.1136/vr.h6830
81	2015	FTP	Full Text (Journal)	UK	19	AHAS	AM	Ge	Yes	English	Pinillos, R.G., Appleby, M.C., Scott-Park, F. and Smith, C.W. (2015), One Welfare. Veterinary Record, 177: 629-630. https://doi.org/10.1136/vr.h6830
82	2017	FTP	Full Text (Journal)	UK	2	AHAS	AM	Ge	Yes	English	Pinillos, R. G. (2017). Consultation to define a One Welfare framework. *The Veterinary Record*, *180*(7), 184.
82	2017	FTP	Full Text (Journal)	UK	2	PGE	LPE	Ge	Yes	English	Pinillos, R. G. (2017). Consultation to define a One Welfare framework. *The Veterinary Record*, *180*(7), 184.

82	2017	FTP	Full Text (Journal)	UK	2	HAB-MH	HAI	Ge	Yes	English	Pinillos, R. G. (2017). Consultation to define a One Welfare framework. *The Veterinary Record*, *180*(7), 184.
82	2017	FTP	Full Text (Journal)	UK	2	AHAS	HAD	Ge	Yes	English	Pinillos, R. G. (2017). Consultation to define a One Welfare framework. *The Veterinary Record*, *180*(7), 184.
82	2017	FTP	Full Text (Journal)	UK	2	SEED	SRM	Ge	Yes	English	Pinillos, R. G. (2017). Consultation to define a One Welfare framework. *The Veterinary Record*, *180*(7), 184.
83	2022	STP	Full Text (Journal)	Canada	8	SEED	TKSI	PR	No	English	Chen, M., & Weary, D. M. (2022). "Cattle Welfare Is Basically Human Welfare": Workers' Perceptions of 'Animal Welfare'on Two Dairies in China. *Frontiers in Veterinary Science*, *8*, 808767.
83	2022	STP	Full Text (Journal)	Canada	8	AHAS	AM	PR	No	English	Chen, M., & Weary, D. M. (2022). "Cattle Welfare Is Basically Human Welfare": Workers' Perceptions of 'Animal Welfare'on Two Dairies in China. *Frontiers in Veterinary Science*, *8*, 808767.
84	2019	STP	Book-chapter	UK	14	AHAS	AM	Ge	Yes	English	Broom, D. M., Johnson, K. G., Broom, D. M., & Johnson, K. G. (2019). One welfare, one health, one stress: humans and other animals. *Stress and Animal Welfare: Key Issues in the Biology of Humans and Other Animals*, 1-13.
84	2019	STP	Book-chapter	UK	14	HAB-MH	HAI	Ge	Yes	English	Broom, D. M., Johnson, K. G., Broom, D. M., & Johnson, K. G. (2019). One welfare, one health, one stress: humans and other animals. *Stress and Animal Welfare: Key Issues in the Biology of Humans and Other Animals*, 1-13.
85	2024	STP	Full Text	USA	2	AHAS	AM	WA	Yes	English	Fischer, B., Pempek, J. A., Flint, J., Wittum, T.,

			(Journal)								& Flint, M. (2024). Application of a One Welfare-Based Ecosystem Model to assess wild collection for public aquariums. *Aquatic Conservation: Marine and Freshwater Ecosystems*, *34*(2), e4098.
85	2024	STP	Full Text (Journal)	USA	2	HAB-MH	HAI	WA	Yes	English	Fischer, B., Pempek, J. A., Flint, J., Wittum, T., & Flint, M. (2024). Application of a One Welfare-Based Ecosystem Model to assess wild collection for public aquariums. *Aquatic Conservation: Marine and Freshwater Ecosystems*, *34*(2), e4098.
85	2024	STP	Full Text (Journal)	USA	2	SEED	SRM	WA	Yes	English	Fischer, B., Pempek, J. A., Flint, J., Wittum, T., & Flint, M. (2024). Application of a One Welfare-Based Ecosystem Model to assess wild collection for public aquariums. *Aquatic Conservation: Marine and Freshwater Ecosystems*, *34*(2), e4098.
85	2024	STP	Full Text (Journal)	USA	2	PGE	LPE	WA	Yes	English	Fischer, B., Pempek, J. A., Flint, J., Wittum, T., & Flint, M. (2024). Application of a One Welfare-Based Ecosystem Model to assess wild collection for public aquariums. *Aquatic Conservation: Marine and Freshwater Ecosystems*, *34*(2), e4098.
85	2024	STP	Full Text (Journal)	USA	2	SEED	TKSI	WA	Yes	English	Fischer, B., Pempek, J. A., Flint, J., Wittum, T., & Flint, M. (2024). Application of a One Welfare-Based Ecosystem Model to assess wild collection for public aquariums. *Aquatic Conservation: Marine and Freshwater Ecosystems*, *34*(2), e4098.
86	2023	STP	Full Text	UK	3	PGE	LPE	PR	No	English	Teixeira, D. L., Salazar, L. C., & Boyle, L. A.

			(Journal)								(2023). The capacity of ante-mortem inspection of pigs at the abattoir to predict post-mortem findings and associated financial implications: A study at batch level. *Animal Science Journal*, *94*(1), e13843.
86	2023	STP	Full Text (Journal)	UK	3	AHAS	AM	PR	No	English	Teixeira, D. L., Salazar, L. C., & Boyle, L. A. (2023). The capacity of ante-mortem inspection of pigs at the abattoir to predict post-mortem findings and associated financial implications: A study at batch level. *Animal Science Journal*, *94*(1), e13843.
87	2023	STP	Full Text (Journal)	France	1	AHAS	AM	Ge	No	French	Ridremont P.B. (2023). UPDATE ON VETERINARY VACCINES: 2. ADAPTATION TO HEALTH SSUES AND SOCIETAL CHALLENGES. Bull. Acad. Vét. France
87	2023	STP	Full Text (Journal)	France	0	AHAS	HAD	Ge	No	French	Ridremont P.B. (2023). UPDATE ON VETERINARY VACCINES: 2. ADAPTATION TO HEALTH SSUES AND SOCIETAL CHALLENGES. Bull. Acad. Vét. France
88	2023	STP	Full Text (Journal)	Italy	1	AHAS	AM	CA	Yes	English	Fossati, P. (2023). Challenges in Sheltering Seized Animals from Hoarders from a One Welfare Perspective. *Animals*, *13*(21), 3303.
88	2023	STP	Full Text (Journal)	Italy	1	HAB-MH	HAI	CA	Yes	English	Fossati, P. (2023). Challenges in Sheltering Seized Animals from Hoarders from a One Welfare Perspective. *Animals*, *13*(21), 3303.
89	2023	STP	Full Text (Journal)	Colombia	1	AHAS	AM	CA	Yes	Spanish	Estepa-Becerra, J. A., Cajiao-Pachón, M. N., & Monsalve-Barrero, S. (2023). Gestión poblacional canina y felina en el marco One Welfare: Una mirada retrospectiva Bogotá 2004 a 2021. *Revista MVZ Córdoba*, *28*(1),

											e2925-e2925.
89	2023	STP	Full Text (Journal)	Colombia	1	HAB-MH	HAI	CA	Yes	Spanish	Estepa-Becerra, J. A., Cajiao-Pachón, M. N., & Monsalve-Barrero, S. (2023). Gestión poblacional canina y felina en el marco One Welfare: Una mirada retrospectiva Bogotá 2004 a 2021. *Revista MVZ Córdoba*, *28*(1), e2925-e2925.
90	2023	STP	Full Text (Journal)	UK	7	AHAS	AM	WS	No	English	Kubasiewicz, L. M., Watson, T., Nye, C., Chamberlain, N., Perumal, R. K., Saroja, R., ... & Burden, F. A. (2023). Bonded labour and donkey ownership in the brick kilns of India: A need for reform of policy and practice. *Animal Welfare*, *32*, e8.
90	2023	STP	Full Text (Journal)	UK	7	HAB-MH	HAI	WS	No	English	Kubasiewicz, L. M., Watson, T., Nye, C., Chamberlain, N., Perumal, R. K., Saroja, R., ... & Burden, F. A. (2023). Bonded labour and donkey ownership in the brick kilns of India: A need for reform of policy and practice. *Animal Welfare*, *32*, e8.
90	2023	STP	Full Text (Journal)	UK	7	PGE	LPE	WS	No	English	Kubasiewicz, L. M., Watson, T., Nye, C., Chamberlain, N., Perumal, R. K., Saroja, R., ... & Burden, F. A. (2023). Bonded labour and donkey ownership in the brick kilns of India: A need for reform of policy and practice. *Animal Welfare*, *32*, e8.
90	2023	STP	Full Text (Journal)	UK	7	SEED	TKSI	WS	No	English	Kubasiewicz, L. M., Watson, T., Nye, C., Chamberlain, N., Perumal, R. K., Saroja, R., ... & Burden, F. A. (2023). Bonded labour and donkey ownership in the brick kilns of India: A need for reform of policy and practice. *Animal

											Welfare, *32*, e8.
91	2023	STP	Full Text (Journal)	Brazil	8	AHAS	AM	PR	Yes	English	Hargreaves-Méndez MJ and Hötzel MJ (2023). A systematic review on whether regenerative agriculture improves animal welfare: A qualitative analysis with a One Welfare perspective. Animal Welfare, 32, e36, 1–12
91	2023	STP	Full Text (Journal)	Brazil	8	PGE	LPE	PR	Yes	English	Hargreaves-Méndez MJ and Hötzel MJ (2023). A systematic review on whether regenerative agriculture improves animal welfare: A qualitative analysis with a One Welfare perspective. Animal Welfare, 32, e36, 1–12
92	2023	STP	Full Text (Journal)	Canada	1	AHAS	AM	WS	No	English	Riley, C. B., Padalino, B., Rogers, C. W., & Thompson, K. R. (2023). Human injuries associated with the transport of horses by road. *Animals*, *13*(10), 1594.
92	2023	STP	Full Text (Journal)	Canada	1	AHAS	HAD	WS	No	English	Riley, C. B., Padalino, B., Rogers, C. W., & Thompson, K. R. (2023). Human injuries associated with the transport of horses by road. *Animals*, *13*(10), 1594.
93	2023	STP	Book-chapter	USA	4	AHAS	AM	CA	Yes	English	Jegatheesan, B., Ormerod, E., Graham, T. M., Stone, W., Power, E. R., Rook, D., & McCune, S. (2023). Pets and Housing: A One Health One Welfare Issue. In *The Routledge International Handbook of Human-Animal Interactions and Anthrozoology* (pp. 109-122). Routledge.
93	2023	STP	Book-chapter	USA	4	HAB-MH	PSY	CA	Yes	English	Jegatheesan, B., Ormerod, E., Graham, T. M., Stone, W., Power, E. R., Rook, D., & McCune,

											S. (2023). Pets and Housing: A One Health One Welfare Issue. In *The Routledge International Handbook of Human-Animal Interactions and Anthrozoology* (pp. 109-122). Routledge.
93	2023	STP	Book-chapter	USA	4	SEED	TKSI	CA	Yes	English	Jegatheesan, B., Ormerod, E., Graham, T. M., Stone, W., Power, E. R., Rook, D., & McCune, S. (2023). Pets and Housing: A One Health One Welfare Issue. In The Routledge International Handbook of Human-Animal Interactions and Anthrozoology (pp. 109-122). Routledge.
93	2023	STP	Book-chapter	USA	4	HAB-MH	HAI	CA	Yes	English	Jegatheesan, B., Ormerod, E., Graham, T. M., Stone, W., Power, E. R., Rook, D., & McCune, S. (2023). Pets and Housing: A One Health One Welfare Issue. In *The Routledge International Handbook of Human-Animal Interactions and Anthrozoology* (pp. 109-122). Routledge.
94	2023	STP	Full Text (Journal)	Australia	2	AHAS	AM	WA	Yes	English	Jones, B., Herbert, C., Finnerty, S., Kennedy, B., Lykins, A., Martin, J. M., ... & McGreevy, P. D. (2023). In Situ Provisioning Wildlife with Food, Water, or Shelter after Bushfires: Using a One Welfare Framework to Guide Responses. *Animals*, *13*(22), 3518.
94	2023	STP	Full Text (Journal)	Australia	2	SEED	SRM	WA	Yes	English	Jones, B., Herbert, C., Finnerty, S., Kennedy, B., Lykins, A., Martin, J. M., ... & McGreevy, P. D. (2023). In Situ Provisioning Wildlife with Food, Water, or Shelter after Bushfires: Using a One Welfare Framework to Guide

											Responses. *Animals*, *13*(22), 3518.
95	2023	STP	Full Text (Journal)	Chile	2	AHAS	AM	PR	Yes	English	Larrondo, C., Guevara, R. D., Calderón-Amor, J., Munoz, C., Cáceres, C., Alvarado, M., ... & Di Pillo, F. (2023). One Welfare: Assessing the Effects of Drought and the COVID-19 Pandemic on Farmers' Well-Being and Their Perception of Goats' Welfare. *Animals*, *13*(20), 3297.
95	2023	STP	Full Text (Journal)	Chile	2	HAB-MH	PSY	PR	Yes	English	Larrondo, C., Guevara, R. D., Calderón-Amor, J., Munoz, C., Cáceres, C., Alvarado, M., ... & Di Pillo, F. (2023). One Welfare: Assessing the Effects of Drought and the COVID-19 Pandemic on Farmers' Well-Being and Their Perception of Goats' Welfare. *Animals*, *13*(20), 3297.
95	2023	STP	Full Text (Journal)	Chile	2	SEED	TKSI	PR	Yes	English	Larrondo, C., Guevara, R. D., Calderón-Amor, J., Munoz, C., Cáceres, C., Alvarado, M., ... & Di Pillo, F. (2023). One Welfare: Assessing the Effects of Drought and the COVID-19 Pandemic on Farmers' Well-Being and Their Perception of Goats' Welfare. Animals, 13(20), 3297.
96	2023	STP	Full Text (Journal)	Colombia	0	AHAS	AM	PR	Yes	Spanish	Medrano-Galarza, C., Ahumada Beltrán, D. G., Zúñiga López, A., Cubides-Cárdenas, J. A., Rojas-Morales, D. M., Albarracín Arias, L. O., ... & García Castro, F. E. (2023). Un Bienestar para todos: Asociaciones entre indicadores de bienestar ovino y la salud mental de productores. *Revista MVZ Córdoba*, *28*(2).

96	2023	STP	Full Text (Journal)	Colombia	0	HAB-MH	PSY	PR	Yes	Spanish	Medrano-Galarza, C., Ahumada Beltrán, D. G., Zúñiga López, A., Cubides-Cárdenas, J. A., Rojas-Morales, D. M., Albarracín Arias, L. O., ... & García Castro, F. E. (2023). Un Bienestar para todos: Asociaciones entre indicadores de bienestar ovino y la salud mental de productores. *Revista MVZ Córdoba*, *28*(2).
97	2023	STP	Full Text (Journal)	Australia	22	AHAS	AM	CA	No	English	McDowall, S., Hazel, S. J., Cobb, M., & Hamilton-Bruce, A. (2023). Understanding the Role of Therapy Dogs in Human Health Promotion. *International Journal of Environmental Research and Public Health*, *20*(10), 5801.
97	2023	STP	Full Text (Journal)	Australia	22	HAB-MH	HAI	CA	No	English	McDowall, S., Hazel, S. J., Cobb, M., & Hamilton-Bruce, A. (2023). Understanding the Role of Therapy Dogs in Human Health Promotion. *International Journal of Environmental Research and Public Health*, *20*(10), 5801.
97	2023	STP	Full Text (Journal)	Australia	22	AHAS	HAD	CA	No	English	McDowall, S., Hazel, S. J., Cobb, M., & Hamilton-Bruce, A. (2023). Understanding the Role of Therapy Dogs in Human Health Promotion. *International Journal of Environmental Research and Public Health*, *20*(10), 5801.
98	2023	STP	Full Text (Journal)	USA	0	AHAS	AM	WA	No	English	Flint, M., & Flint, J. (2023). Use of soybean as an alternative protein source for welfare-orientated production of American alligators (Alligator mississippiensis). *PeerJ*, *11*, e16321.

98	2023	STP	Full Text (Journal)	USA	0	SEED	SRM	WA	No	English	Flint, M., & Flint, J. (2023). Use of soybean as an alternative protein source for welfare-orientated production of American alligators (Alligator mississippiensis). *PeerJ*, *11*, e16321.
99	2024	STP	Full Text (Journal)	UK	17	AHAS	AM	WS	No	English	Vasanthakumar, M.A.; Upjohn, M.M.; Watson, T.L.; Dwyer, C.M. 'All My Animals Are Equal, but None Can Survive without the Horse'.The Contribution of Working Equids to the Livelihoods of Women across Six Communities in the Chimaltenango Region of Guatemala. Animals 2021, 11, 1509.
99	2024	STP	Full Text (Journal)	UK	17	HAB-MH	HAI	WS	No	English	Vasanthakumar, M.A.; Upjohn, M.M.; Watson, T.L.; Dwyer, C.M. 'All My Animals Are Equal, but None Can Survive without the Horse'.The Contribution of Working Equids to the Livelihoods of Women across Six Communities in the Chimaltenango Region of Guatemala. Animals 2021, 11, 1509.
99	2024	STP	Full Text (Journal)	UK	17	SEED	TKSI	WS	No	English	Vasanthakumar, M.A.; Upjohn, M.M.; Watson, T.L.; Dwyer, C.M. 'All My Animals Are Equal, but None Can Survive without the Horse'.The Contribution of Working Equids to the Livelihoods of Women across Six Communities in the Chimaltenango Region of Guatemala. Animals 2021, 11, 1509.
100	2024	STP	Full Text (Journal)	Australia	13	HAB-MH	HAI	CA	No	English	Cotterell, J.L.; Rand, J.; Barnes, T.S.; Scotney, R. Impact of a Local Government Funded Free Cat Sterilization Program for Owned and Semi-Owned Cats. Animals 2024, 14,1615.

											ttps://doi.org/10.3390/ ani14111615
100	2024	STP	Full Text (Journal)	Australia	13	AHAS	AM	CA	No	English	Cotterell, J.L.; Rand, J.; Barnes, T.S.; Scotney, R. Impact of a Local Government Funded Free Cat
100	2024	STP	Full Text (Journal)	Australia	13	PGE	LPE	CA	No	English	Cotterell, J.L.; Rand, J.; Barnes, T.S.; Scotney, R. Impact of a Local Government Funded Free Cat
101	2024	STP	Full Text (Journal)	South Africa	1	HAB-MH	HAI	WA	Yes	English	Van de Wat, A., Garaï, M. E., Burnett, M. M., Henley, M. D., Di Minin, E., Streicher, J. P., ... & Slotow, R. (2024). Integrating a "One Well-being" approach in elephant conservation: evaluating consequences of management interventions. Ecology and Society, 29(3), 15.
101	2024	STP	Full Text (Journal)	South Africa	1	AHAS	AM	WA	Yes	English	Van de Wat, A., Garaï, M. E., Burnett, M. M., Henley, M. D., Di Minin, E., Streicher, J. P., ... & Slotow, R. (2024). Integrating a "One Well-being" approach in elephant conservation: evaluating consequences of management interventions. Ecology and Society, 29(3), 15.
101	2024	STP	Full Text (Journal)	South Africa	1	PGE	LPE	WA	Yes	English	Van de Wat, A., Garaï, M. E., Burnett, M. M., Henley, M. D., Di Minin, E., Streicher, J. P., ... & Slotow, R. (2024). Integrating a "One Well-being" approach in elephant conservation: evaluating consequences of management interventions. Ecology and Society, 29(3), 15.
101	2024	STP	Full Text (Journal)	South Africa	1	SEED	SRM	WA	Yes	English	Van de Wat, A., Garaï, M. E., Burnett, M. M., Henley, M. D., Di Minin, E., Streicher, J. P., ... & Slotow, R. (2024). Integrating a "One Well-being" approach in elephant conservation:

											evaluating consequences of management interventions. Ecology and Society, 29(3), 15.
102	2024	STP	Full Text (Journal)	France	1	PGE	LPE	Ge	No	French	Angot, J. L., & Rosolen, S. G. (2024). Bien-être et bientraitance des animaux. Bulletin de l'Académie Nationale de Médecine, 208(3), 283-289.
102	2024	STP	Full Text (Journal)	France	0	AHAS	AM	Ge	No	French	Angot, J. L., & Rosolen, S. G. (2024). Bien-être et bientraitance des animaux. Bulletin de l'Académie Nationale de Médecine, 208(3), 283-289.
103	2024	STP	Book-chapter	UK	3	AHAS	AM	PR	No	English	Broom, D. M. One biology, sustainable and regenerative farming: A role for pig and poultry production?. In *Regenerative Farming and Sustainable Diets* (pp. 107-115). Routledge.
103	2024	STP	Book-chapter	UK	3	SEED	SRM	PR	No	English	Broom, D. M. One biology, sustainable and regenerative farming: A role for pig and poultry production?. In *Regenerative Farming and Sustainable Diets* (pp. 107-115). Routledge.
104	2024	STP	Full Text (Journal)	USA	1	AHAS	AM	CA	No	English	Pearce, H. L., Spielman, B., Weatherwax, C., & Pairis-Garcia, M. (2024). A novel corporate-community partnership model provides safe housing for pets of domestic violence victims. Journal of the American Veterinary Medical Association, 262(5), 685-691.
104	2024	STP	Full Text (Journal)	USA	1	HAB-MH	HAI	CA	No	English	Pearce, H. L., Spielman, B., Weatherwax, C., & Pairis-Garcia, M. (2024). A novel corporate-community partnership model provides safe housing for pets of domestic violence victims.

											Journal of the American Veterinary Medical Association, 262(5), 685-691.
104	2024	STP	Full Text (Journal)	USA	1	HAB-MH	PSY	CA	No	English	Pearce, H. L., Spielman, B., Weatherwax, C., & Pairis-Garcia, M. (2024). A novel corporate-community partnership model provides safe housing for pets of domestic violence victims. Journal of the American Veterinary Medical Association, 262(5), 685-691.
104	2024	STP	Full Text (Journal)	USA	1	SEED	TKSI	CA	No	English	Pearce, H. L., Spielman, B., Weatherwax, C., & Pairis-Garcia, M. (2024). A novel corporate-community partnership model provides safe housing for pets of domestic violence victims. Journal of the American Veterinary Medical Association, 262(5), 685-691.
105	2024	STP	Full Text (Journal)	Norway	2	SEED	TKSI	PR	No	English	Steen, N. A., Muri, K., & Torske, M. O. (2024). Exploring longitudinal associations between farmer wellbeing and the welfare of their livestock. The HUNT Study, Norway. Preventive Veterinary Medicine, 233, 106361.
105	2024	STP	Full Text (Journal)	Norway	2	AHAS	AM	PR	No	English	Steen, N. A., Muri, K., & Torske, M. O. (2024). Exploring longitudinal associations between farmer wellbeing and the welfare of their livestock. The HUNT Study, Norway. Preventive Veterinary Medicine, 233, 106361.
106	2024	STP	Full Text (Journal)	Chile	0	AHAS	AM	CA	Yes	Spanish	San Martín, E. L. E. C., Echeverría-Jaque, C. L., & Macuer-Guzmán, J. E. (2024). Análisis de la relación entre la obesidad de tutores, sus perros y One Welfare. Una Revisión bibliográfica. European Public & Social Innovation Review, 9, 1-17.

106	2024	STP	Full Text (Journal)	Chile	0	HAB-MH	HAI	CA	Yes	Spanish	San Martín, E. L. E. C., Echeverría-Jaque, C. L., & Macuer-Guzmán, J. E. (2024). Análisis de la relación entre la obesidad de tutores, sus perros y One Welfare. Una Revisión bibliográfica. European Public & Social Innovation Review, 9, 1-17.
106	2024	STP	Full Text (Journal)	Chile	0	AHAS	HAD	CA	Yes	Spanish	San Martín, E. L. E. C., Echeverría-Jaque, C. L., & Macuer-Guzmán, J. E. (2024). Análisis de la relación entre la obesidad de tutores, sus perros y One Welfare. Una Revisión bibliográfica. European Public & Social Innovation Review, 9, 1-17.
107	2024	STP	Full Text (Journal)	Romania	0	AHAS	AM	PR	Yes	English	URDES, L., WALSTER, C., TEPPER, J., & FOYLE, L. (2024). HOW ONE HEALTH AND ONE WELFARE CAN STRENGTHEN THE EVIDENCE OF A MANAGEMENT PROCEDURE-A CASE STUDY OF EYESTALK ABLATION IN FARMED SHRIMP. Scientific Papers. Series D. Animal Science, 67(1).
108	2024	STP	Full Text (Journal)	Colombia	0	AHAS	AM	PR	Yes	English	Romero, M. H., Gallego-Polania, S. A., & Sanchez, J. A. (2024). Natural Savannah Systems Within the “One Welfare” Approach: Part 1. Traditional Farmers' Perspectives, Environmental Challenges and Opportunities.
108	2024	STP	Full Text (Journal)	Colombia	0	SEED	TKSI	PR	Yes	English	Romero, M. H., Gallego-Polania, S. A., & Sanchez, J. A. (2024). Natural Savannah Systems Within the “One Welfare” Approach: Part 2. Traditional Farmers' Perspectives, Environmental Challenges and Opportunities.
108	2024	STP	Full Text	Colombia	0	SEED	SRM	PR	Yes	English	Romero, M. H., Gallego-Polania, S. A., &

			(Journal)									Sanchez, J. A. (2024). Natural Savannah Systems Within the “One Welfare” Approach: Part 3. Traditional Farmers' Perspectives, Environmental Challenges and Opportunities.
108	2024	STP	Full Text (Journal)	Colombia	0	PGE	LPE	PR	Yes	English		Romero, M. H., Gallego-Polania, S. A., & Sanchez, J. A. (2024). Natural Savannah Systems Within the “One Welfare” Approach: Part 4. Traditional Farmers' Perspectives, Environmental Challenges and Opportunities.
109	2024	STP	Full Text (Website)	Australia	1	AHAS	AM	CA	Yes	English		Clay, L. (2024). One Welfare Approach: Wellbeing in Shelters. Today's Veterinary Nurse, Spring 2024, 59.
109	2024	STP	Full Text (Website)	Australia	1	HAB-MH	HAI	CA	Yes	English		Clay, L. (2024). One Welfare Approach: Wellbeing in Shelters. Today's Veterinary Nurse, Spring 2024, 59.
110	2023	STP	Book - chapter	Denmark	9	AHAS	AM	PR	Yes	English		Lena Lidfors, Bente Berget, Karen Thodberg (2023). Farm-Based Interventions Considering One Health – One Welfare. In The Routledge International Handbook of Human-Animal Interactions and Anthrozoology, Edited by Aubrey H. Fine, Megan K. Mueller, Zenithson Y. Ng, Alan M. Beck, Jose M. Peralta, ImprintRoutledge.
110	2023	STP	Book - chapter	Denmark	9	HAB-MH	HAI	PR	Yes	English		Lena Lidfors, Bente Berget, Karen Thodberg (2023). Farm-Based Interventions Considering One Health – One Welfare. In The Routledge International Handbook of Human-Animal Interactions and Anthrozoology, Edited by Aubrey H. Fine, Megan K. Mueller, Zenithson Y. Ng, Alan M. Beck, Jose M. Peralta,

											ImprintRoutledge.
111	2020	STP	Book - chapter	USA	0	HAB-MH	HAI	CA	Yes	English	Jegatheesan B., Ormerod E. (2020). The human-animal bond and one health, one welfare. In Animal-Assisted Interventions for Health and Human Service Professionals, edited by Carlie J. Driscoll, Nova Science Publishers, pp. 3 - 33,
111	2020	STP	Book - chapter	USA	0	HAB-MH	Psy	CA	Yes	English	Jegatheesan B., Ormerod E. (2020). The human-animal bond and one health, one welfare. In Animal-Assisted Interventions for Health and Human Service Professionals, edited by Carlie J. Driscoll, Nova Science Publishers, pp. 3 - 33,
